# Evaluation of Histone Deacetylase Inhibitors as Radiosensitizers for Proton and Light Ion Radiotherapy

**DOI:** 10.3389/fonc.2021.735940

**Published:** 2021-08-26

**Authors:** Alicia M. Johnson, Paula V. Bennett, Katherine Z. Sanidad, Anthony Hoang, James H. Jardine, Deborah J. Keszenman, Paul F. Wilson

**Affiliations:** ^1^Biology Department, Brookhaven National Laboratory, Upton, NY, United States; ^2^Laboratorio de Radiobiología Médica y Ambiental, Grupo de Biofisicoquímica, Centro Universitario Regional Litoral Norte, Universidad de la República (UdelaR), Salto, Uruguay; ^3^Department of Radiation Oncology, University of California–Davis, Sacramento, CA, United States

**Keywords:** HDAC inhibitor, SAHA, radiosensitization, double-strand break, ionizing radiation, protons, carbon ions, transformation

## Abstract

Significant opportunities remain for pharmacologically enhancing the clinical effectiveness of proton and carbon ion-based radiotherapies to achieve both tumor cell radiosensitization and normal tissue radioprotection. We investigated whether pretreatment with the hydroxamate-based histone deacetylase inhibitors (HDACi) SAHA (vorinostat), M344, and PTACH impacts radiation-induced DNA double-strand break (DSB) induction and repair, cell killing, and transformation (acquisition of anchorage-independent growth in soft agar) in human normal and tumor cell lines following gamma ray and light ion irradiation. Treatment of normal NFF28 primary fibroblasts and U2OS osteosarcoma, A549 lung carcinoma, and U87MG glioma cells with 5–10 µM HDACi concentrations 18 h prior to cesium-137 gamma irradiation resulted in radiosensitization measured by clonogenic survival assays and increased levels of colocalized gamma-H2AX/53BP1 foci induction. We similarly tested these HDACi following irradiation with 200 MeV protons, 290 MeV/n carbon ions, and 350 MeV/n oxygen ions delivered in the Bragg plateau region. Unlike uniform gamma ray radiosensitization, effects of HDACi pretreatment were unexpectedly cell type and ion species-dependent with C-12 and O-16 ion irradiations showing enhanced G0/G1-phase fibroblast survival (radioprotection) and in some cases reduced or absent tumor cell radiosensitization. DSB-associated foci levels were similar for proton-irradiated DMSO control and SAHA-treated fibroblast cultures, while lower levels of induced foci were observed in SAHA-pretreated C-12 ion-irradiated fibroblasts. Fibroblast transformation frequencies measured for all radiation types were generally LET-dependent and lowest following proton irradiation; however, both gamma and proton exposures showed hyperlinear transformation induction at low doses (≤25 cGy). HDACi pretreatments led to overall lower transformation frequencies at low doses for all radiation types except O-16 ions but generally led to higher transformation frequencies at higher doses (>50 cGy). The results of these *in vitro* studies cast doubt on the clinical efficacy of using HDACi as radiosensitizers for light ion-based hadron radiotherapy given the mixed results on their radiosensitization effectiveness and related possibility of increased second cancer induction.

## Introduction

Despite the improved dose distributions and increased relative biological effectiveness (RBE) afforded by accelerated proton and carbon ion-based hadron radiotherapies (1), significant opportunities remain for enhancing their clinical effectiveness through pharmacological means to achieve tumor cell radiosensitization and normal tissue radioprotection. Identifying effective radiosensitizers that synergistically enhance charged particle-induced tumor cell killing would allow for lower doses per fraction to be used, thereby reducing normal tissue exposures. Alternatively, an increased therapeutic index could be achieved using charged particle normal tissue radioprotectors that would allow for boosting of tumor doses (assuming the agent does not likewise radioprotect tumor cells). Charged particles have been shown to induce higher relative frequencies of closely localized DNA double-strand breaks (DSBs) and clustered DNA damages along their tracks compared to low linear energy transfer (LET) photon radiations (2, 3). This feature underlies their higher RBE for cell killing *in vitro* and tumor control *in vivo* (4). The density and complexity of these lesions activate several cellular DNA damage response (DDR) pathways, and their repair and restitution require the participation of multiple DNA signaling and repair pathways to properly identify them and coordinate their repair as cells progress through the cell cycle (5–7). Compared to low LET X- and γ-rays, it has been shown the repair of intermediate to high LET HZE ion-induced DNA damage requires relatively more reliance on homologous recombinational repair (HRR) as damages that were not repaired in G0/G1 phase by non-homologous end-joining (NHEJ) and base excision repair (BER) present themselves during subsequent chromatin decondensation and DNA replication in S phase (8–11). Exposures to charged particles, including low LET protons, are consistently associated with higher relative induction of DSB-associated foci, prematurely condensed chromosomal breaks, and simple and complex chromosomal aberrations measured *in vitro* post-irradiation compared to X- and γ-rays (9, 12–15).

Targeting the charged particle-induced DDR offers multiple promising radiosensitization approaches for hadron radiotherapies by directly inhibiting DNA damage signaling and repair pathways using, *e.g*., PARP, ATM, and DNA-PK-specific inhibitors, or by treating cells with agents that modulate chromatin compaction and epigenetic status such as histone deacetylase inhibitors (HDACi) (16, 17). HDACi prevent the deacetylation of lysines on histone tails by different HDACs by directly binding their catalytic site, functionally maintaining chromatin in a hyperacetylated state that results in decondensation and altered rates of gene expression (18, 19). They have been shown to likewise affect the expression and acetylation status of a number of non-histone nuclear and cytoplasmic proteins involved in DNA repair, apoptosis induction, cell cycle progression, proliferation, and differentiation (20). The HDACi SAHA (suberoylanilide hydroxamic acid; vorinostat), M344 (4-(dimethylamino)-N-[7-(hydroxyamino)-7-oxoheptyl]-benzamide), and PTACH (S-[6-(4-phenyl-2-thiazolylcarbamoyl)hexyl] thioisobutyrate) are hydroxamate analogues that effectively inhibit both class I and II HDACs at nanomolar concentrations, but not other HDAC classes (21). SAHA is US FDA-approved as a stand-alone treatment for cutaneous T-cell lymphoma in patients with progressive or recurrent disease on or following two systemic therapies (22) and shows promise for the treatment of other hematological and solid malignancies alone or in combination therapies (23, 24). Patients receiving 200–600 mg vorinostat either orally or intravenously reach plasma/serum concentrations of ~1–2 µM approximately 1–2.5 h following administration (25–27). In (28), 5 µM SAHA treatments of HFS normal human fibroblasts, A549 lung carcinoma, and LNCaP prostate cancer cells *in vitro* resulted in γ-H2AX foci formation, indicating HDACi are capable of inducing DSBs. DSBs were effectively repaired in the normal cells during continuous culture with SAHA and after washout; however, both tumor cell lines demonstrated a persistence of foci and decreased expression of several key DNA damage signaling and repair proteins including Rad50, Mre11, and ATM. HDACs and other acetyltransferases also impact HRR and cellular radiosensitivity by modulating acetylation status and activity of HRR proteins directly. A report by (29) demonstrated the acetylation of Rad52 on multiple sites by p300/CBP acetyltransferase and their impact on Rad52 foci formation, a key step for Rad51 recruitment to IR-induced DSBs. Increased SAHA-mediated cytotoxicity was observed by (30) in U251tk glioblastoma cells expressing herpes simplex thymidine kinase exposed to the nucleoside analogue ganciclovir. In cells treated with 0.3–20 µM SAHA, Rad51 expression was significantly reduced in a concentration-dependent manner, and Rad51 foci formation was nearly completely inhibited at sites of replication-associated DSBs following two cycles of ganciclovir incorporation.

Pretreatments with SAHA and other hydroxamate-based “pan-HDAC” inhibitors such as M344 and panobinostat have been shown to effectively sensitize human tumor cells *in vitro* to X- and γ-rays and *in vivo* in various chemotherapy and radiotherapy clinical trials (20, 21, 31–33). Mechanisms by which HDACi radiosensitize tumor cells include increasing apoptosis induction, altering relative cell cycle distributions, and downregulating the expression of key NHEJ and HRR repair genes. In (34), SAHA pretreatments of DU145 prostate cancer and U373vIII glioma cells (1 and 0.75 µM, respectively) synergistically increased cell killing and apoptosis induction and reduced Rad51 and DNA-PK expression post-IR in 6 Gy-irradiated DU145 cells. This was likewise observed in (31) in which SAHA-mediated radiosensitization of SAOS2 and KHOS-24S osteosarcoma and RD and A-204 rhabdomyosarcoma cell lines was associated with reduced Rad51 and Ku80 expression, increased histone H3 acetylation, and higher levels of both G2/M-phase cell cycle arrest and apoptosis induction following X-irradiation. Interestingly, in this same report, 24 h pretreatment with 1 µM SAHA did not affect the survival of normal human osteoblasts (hFOB 1.19) or diploid fibroblasts (NHDFc).

Fewer reports exist on the responses of human normal and tumor cells treated with HDACi and exposed to accelerated protons or C-12 ions at energies typically employed in hadron radiotherapy. A report by (35) showed increased C-12 ion-induced cell killing and apoptosis, along with higher expression of p21 and γ-H2AX and proportions of G2/M-phase arrested cells, in KHOS-24S osteosarcoma and A-204 rhabdomyosarcoma cells pretreated with 0.5–1 µM SAHA. A radioprotective effect of 1 µM SAHA pretreatment was seen for these same endpoints measured in C-12 ion-irradiated hFOB 1.10 osteoblasts. In (36), 24 h pretreatment of human glioblastoma LN18 and U251 cell lines with 0.5 µM SAHA prior to irradiation with 250 kVp X-rays or 290 MeV/n SOBP C-12 ions resulted in delayed kinetics of γ-H2AX foci resolution and increased cell killing with sensitizer enhancement ratios (SER) of ~1.2–1.55 reported. In (37), 3 h pretreatment with 1 mM valproic acid (VPA) sensitized hepatocellular carcinoma (HCC) cells to 230 MeV SOBP proton-induced cell killing and apoptosis induction. In (38), 24 h pretreatment with 0.2 or 2 µM SAHA sensitized A549 lung carcinoma cells to cesium-137 γ-rays, 200 MeV SOBP protons, and 290 MeV/n SOBP C-12 ions, but did not radiosensitize log-phase or quiescent G0/G1-phase normal AG01522 fibroblasts (aside from perhaps a modest degree of proton radiosensitization). Pretreatment with 2 µM SAHA also significantly reduced Rad51 and RPA foci formation in γ-ray and proton-irradiated A549 cells. Results of these studies suggest a greater potential for HDACi-mediated charged particle tumor cell radiosensitization given the greater reliance on HRR for repairing charged particle-induced clustered DNA damage.

We hypothesized that HDACi pretreatment would lead to effective *in vitro* charged particle radiosensitization of human normal and tumor cell lines similar to (or greater than) levels achieved following X- and γ-ray irradiation. We focused a portion of our studies on the radiation responses of non-transformed cells using low-passage NFF28 primary human fibroblasts as a model normal cell type, given fibroblasts are primary constituents of both normal tissue stroma and tumor microenvironments and contribute significantly to tissue-level IR responses through paracrine and endocrine signaling mechanisms (39). We first identified the most effective concentrations of SAHA, M344, and PTACH for cesium-137 γ-ray radiosensitization of quiescent G0/G1-phase NFF28 fibroblasts and asynchronously growing A549 lung carcinoma, U2OS osteosarcoma, and U87MG malignant glioma cells using single-cell colony formation assays. We then investigated impacts of HDACi pretreatment on cell killing following irradiation with accelerated 200 MeV protons, 290 MeV/n C-12 ions, and 350 MeV/n O-16 ions in the same cell strain/lines, with DSB-associated γ-H2AX/53BP1 foci assays conducted with quiescent NFF28 fibroblasts and cellular transformation (acquisition of anchorage-independent growth in soft agar) assays conducted with asynchronously growing NFF28 cultures. Charged particle irradiations were conducted in the initial entrance/plateau region of the charged particle Bragg curves, as opposed to the much higher relative LET (and associated RBE) portions of the Bragg peak region typically used for clinical hadron radiotherapies (40–43), as these more specifically relate to clinically relevant normal tissue exposures in proximal entrance regions prior to SOBP-targeted tumor volumes.

## Materials and Methods

### Cell Culture and Irradiations

Cultures of NFF28 normal diploid primary human fibroblasts (passage levels 3–8), A549 lung carcinoma (CRL-CCM-185), U2OS osteosarcoma (HTB-96), and U87MG (HTB-14) malignant glioma cells were used in these experiments. The tumor cell lines were obtained from the American Type Culture Collection (ATCC). Strain NFF28 was originally isolated from neonatal foreskin by Dr. Betsy Sutherland’s laboratory at Brookhaven National Laboratory (BNL) (44), and photon and charged particle radiation survival and transformation datasets are available for this fibroblast strain as reported previously (45–48). Cells were grown in αMEM medium supplemented with 15% fetal bovine serum (Hyclone), 100 U/ml penicillin, 100 µg/ml streptomycin, vitamins, amino acids, and GlutaMAX™-I (GIBCO/Invitrogen) in a 37°C incubator supplied with 95% air/5% CO_2_ in standard T-25 tissue culture flasks or Nalge-Nunc™ flaskettes. Cells were maintained in asynchronous log-phase growth for survival and transformation assays or allowed to reach density-inhibited confluence (quiescence) for assessing NFF28 G0/G1-phase survival and DSB-associated foci induction and repair kinetics. For foci assays, NFF28 cells were passaged regularly prior to reaching confluency, seeded at ~30% density into microscope flaskettes (Nalge-Nunc™), and grown for 5 days to full confluency with a medium change on day 2. Suberoylanilide hydroxamic acid (SAHA), 4-(dimethylamino)-N-[7-(hydroxyamino)-7-oxoheptyl]-benzamide (M344), and S-[6-(4-phenyl-2-thiazolylcarbamoyl)hexyl] thioisobutyrate (PTACH) were purchased from Sigma-Aldrich and prepared as 10 or 100 mM stocks dissolved in DMSO. Cultures were pretreated with 1–20 µM concentrations of HDACi or 0.1% DMSO as a vehicle control for 18 h prior to irradiation.

Cesium-137 662-keV γ-ray irradiations were conducted using the BNL Biology Department’s J.L. Shepherd Mark I Model 68A cabinet irradiator at a dose-rate of ~0.7 Gy/min (dosimetry verified by J.L. Shepherd). Charged particle irradiations were conducted at the NASA Space Radiation Laboratory (NSRL) at BNL. Accelerated beams of 200 MeV protons (LET = 0.45 keV/µm in H_2_O), 290 MeV/n C-12 ions (LET = 13.02 keV/µm), and 350 MeV/n O-16 ions (LET = 20.90 keV/µm) were delivered in the entrance/plateau region of the Bragg curve (as opposed to the higher LET Bragg peak or as a SOBP) at dose-rates of ~0.1–0.6 Gy/h. Doses were confirmed by NSRL physicists at the flask position using a NIST-traceable tissue-equivalent ion chamber (EG&G model IC-17) used to calibrate a series of custom parallel-plate beamline ionization chambers to control beam delivery (49). Cell cultures were irradiated at room temperature in sealed flasks or microscope chamber slides. After irradiation, cells were subcultured and plated immediately for survival, or the medium changed and cells incubated at 37°C until subculture for transformation assays. For measurements of DSB-associated foci levels, fibroblast cultures in flaskettes were returned to the NSRL 37°C incubator until collection/fixation from 10 min to 24 h post-irradiation. Sham-irradiated cells for all assays were harvested by identical procedures.

### Immunocytochemistry and DSB-Associated γ-H2AX pS139/53BP1 Foci Imaging/Scoring

DSB-associated colocalized γ-H2AX/53BP1 foci formation was evaluated as described previously (50, 51). The medium in SAHA-treated cultures was not changed post-irradiation, so cells were treated continuously with 10 µM SAHA until being collected at the following time points. At 10 min, 30 min, 2 h, 6 h, and 24 h post-irradiation, flaskettes were removed from the 37°C incubator, rinsed with PBS, and fixed with fresh 4% formaldehyde at room temperature for 15 min. Slides were then permeabilized with 0.5% Triton X-100 on ice for 10 min, and washed with PBS. Slides were blocked in PBS with 1% BSA, 2% fetal bovine and goat sera, 0.1% Triton X-100, and 0.05% Tween-20 for 30 min at room temperature followed by incubation in a 37°C humid chamber for 30 min with one of the following primary antibody combinations diluted 1:400 in PBS with 1% BSA: mouse monoclonal anti-γ-H2AX pS139 (JBW301, Upstate/Millipore) and rabbit polyclonal anti-53BP1 (NB100-304, Novus Biologicals). Slides were rinsed in PBS and incubated in a 37°C humid chamber for 30 min with Alexa Fluor^®^ 488 and 594-conjugated goat anti-mouse and anti-rabbit F(ab′)2 fragments (Molecular Probes/Invitrogen) diluted 1:500 in PBS. Slides were rinsed in PBS, treated with 3.7% formaldehyde to immobilize the antibodies at their target locations, and mounted with ProLong Gold antifade reagent with 0.2 µg/ml 4′, 6-diamidino-2-phenylindole (DAPI; Molecular Probes/Invitrogen). Images were captured using a Zeiss Axio Observer Z1 epifluorescence microscope equipped with a 63× oil immersion objective and appropriate filter sets, and processed using Zeiss AxioVision image analysis software. For two to three independent experiments, ≥50 cells per time-point were scored by eye by two cross-corroborated scorers to minimize any observer bias. Nuclei of atypical size or morphology or those with very high foci counts (*e.g.*, S-phase cells) were not scored.

### Survival and Transformation Assays

Post-irradiation cell survival was determined by single-cell colony formation assays as described previously (52). Immediately following irradiation, appropriate numbers of cells were plated in triplicate in 10-cm culture dishes or T-25 flasks with complete medium to yield ~50–100 viable colonies after 14 days of undisturbed growth at 37°C. Dishes were then aspirated and rinsed with PBS, fixed with 95% ethanol, and stained with a solution of 50% (v/v) Kopykake blue food dye (Kopykake Enterprises), 40% methanol, and 10% glacial acetic acid. Colonies of ≥50 viable cells were scored as survivors. Post-irradiation transformation frequencies were assessed by measurement of anchorage-independent (A-I) growth in soft agar, a selective condition in which untransformed fibroblasts do not proliferate, per established Sutherland lab protocols (45, 47, 53). For A-I growth, 8 ml of agar mix consisting of 80 ml 1.25% Difco Bacto agar, 80 ml 2X DMEM, 20 ml iron-supplemented FCS, 20 ml Difco tryptose phosphate broth (29.5 g/l ddH_2_0) was aliquoted in 60-mm culture dishes and allowed to set. They were then overlaid with a mixture of 1 ml of medium plus serum containing 10^5^ cells plus 2 ml agar mix and allowed to solidify at RT. Plates were incubated at 37°C for 20 days with weekly medium changes to maintain proper humidity, after which A-I colonies of ≥50 cells were counted under a dissecting microscope. The frequency of transformants per survivor was determined from the ratio of A-I colonies per number of surviving cells plated, with ≥6 replicate dishes counted per data point in at least two independent experiments.

### Statistical Analyses

Statistical analyses were performed using Prism (GraphPad Software). Survival data were fit using Prism’s weighted least-squares linear-quadratic survival curve-fitting function; survival datasets were best fit by either a linear-quadratic (LQ; S = e^–αD– βD^2^) or linear (S = e^–αD^) exponential function. Transformation data were best fit by Prism using weighted least-squares regression hyperbolic curve fitting over the full dose range and linearly for low doses ≤25 cGy. Low dose DSB-associated foci induction data at low doses were also fitted by linear regression curve fits. Statistical differences among datasets were tested using unpaired two-tailed Student’s *t*-tests with Welch’s correction and one-way or two-way ANOVA with Tukey’s HSD *post hoc* test (Prism) used to identify statistically significant differences among groups; *p*-values ≤0.05 were considered statistically significant differences.

## Results

### Gamma Ray Survival Assays

**Figure 1:**
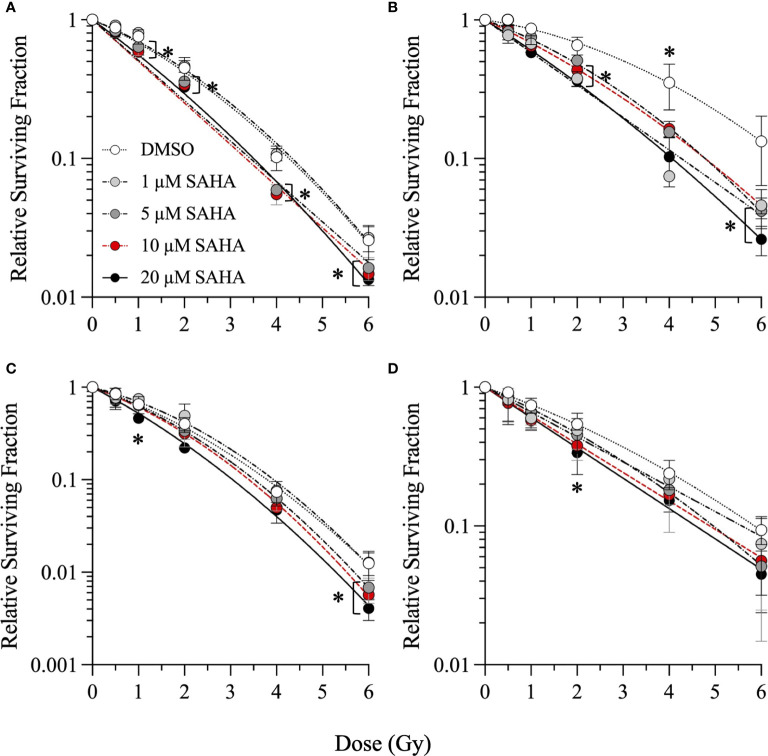
Clonogenic survival of G0/G1-phase normal NFF28 primary fibroblasts **(A)** and asynchronously growing A549 lung carcinoma **(B)**, U2OS osteosarcoma **(C)**, and U87MG malignant glioma cells **(D)** pretreated for 18 h with 1–20 µM SAHA and exposed to 0.5–6 Gy cesium-137 γ-rays (LET = 0.91 keV/µm). Data reported as mean ± SD; where error bars are not visible, they are smaller than the data point. Asterisks mark significant differences compared to DMSO controls at *p*-values of ≤0.05 (*) by one-way ANOVA.

Single-cell colony formation assays were first performed with 1–20 µM concentrations of SAHA, M344, and PTACH to determine their optimum concentrations for cesium-137 γ-ray radiosensitization of NFF28 fibroblasts and A549 lung carcinoma, U2OS osteosarcoma, and U87MG malignant glioma cells. Sham-irradiated plating (cloning) efficiencies (PE) for 1–20 µM HDACi 18 h pretreatments for all four cell types are shown in **Supplementary Figure 1**. Quiescent G0/G1-phase NFF28 fibroblasts showed increased PE values following pretreatment with all three HDACi at the concentrations tested, and the three tumor lines generally showing reduced plating efficiencies with increasing inhibitor concentrations. Survival curves for NFF28, A549, U2OS, and U87MG cells irradiated with 0.5–6 Gy γ-rays are shown in **Figure 1** for cultures pretreated with SAHA and **Supplementary Figures 2**, **3** for cultures pretreated with M344 and PTACH, respectively. Significant radiosensitization of NFF28 fibroblasts was observed for all three HDACi at higher doses of 1–6 Gy (*p* = <0.0001–0.0221 for SAHA, 0.0008–0.0457 for M344, 0.0009–0.0161 for PTACH by one-way ANOVA). Of the three HDACi tested, SAHA provided the highest degree of γ-ray radiosensitization for the three tumor lines (*p* = 0.0037–0.0406 for A549 for doses of 2–6 Gy, 0.0018–0.0317 for U2OS at 6 Gy, and 0.0466 for 2 Gy-irradiated U87MG cells pretreated with 20 µM SAHA). M344 was only effective at significantly radiosensitizing A549 cells at 2 Gy only (10 µM M344; *p* = 0.0202) and U2OS and U87MG cells at the highest dose of 6 Gy (*p*=0.0013–0.0027 for 5–20 µM M344 and *p*=0.0293 and 0.0374 for 10 and 20 µM M344, respectively). The results of the γ-ray clonogenic survival assays are summarized in **Supplementary Table 1** with D_10_ (doses required to reduce relative survival to 10%) and SER values (calculated as the ratios of D_10_ values of HDACi-pretreated cells to DMSO vehicle controls) reported.

SER values for 1–20 µM concentrations of the three HDACi are shown in **Supplementary Figure 4** with levels of γ-ray radiosensitization in the four cell types generally increasing in a concentration-dependent manner. A549 lung carcinoma cells showed the greatest degree of radiosensitization with SER values of 1.36–1.6 following 1–20 µM SAHA pretreatment (*p* = 0.0359 and 0.0133 for 1 and 20 µM SAHA, respectively, by two-way ANOVA), along with values of 1.32 and 1.16 for 10 µM M344 and 5 µM PTACH, respectively. SER values ranged from 1.16 to 1.33 for quiescent NFF28 fibroblasts, 1.03–1.25 for U2OS osteosarcoma cells and 1.08–1.31 for U87MG cells (*p* = 0.0065 for 20 µM SAHA and 0.0072 for 10 µM M344). Both SAHA and M344 showed more efficient γ-ray radiosensitization than PTACH in all three tumor cell lines, while all three compounds equally radiosensitized NFF28 fibroblasts. Interestingly, slightly increased survival (*i.e.*, mild radioprotection) was observed for quiescent NFF28 fibroblasts pretreated with 1 µM concentrations of all three HDACi and also for U2OS osteosarcoma cells pretreated with 1 µM SAHA and PTACH (both are mesenchymal-derived cell types). Based on results of these assays, we chose 10 µM SAHA, 10 µM M344, and 5 µM PTACH as the most effective radiosensitizer concentrations (that did not likewise appreciably decrease PE per **Supplementary Figure 1**) for the subsequent charged particle irradiations.

### Proton, Carbon, and Oxygen Ion Survival Assays

**Figure 2:**
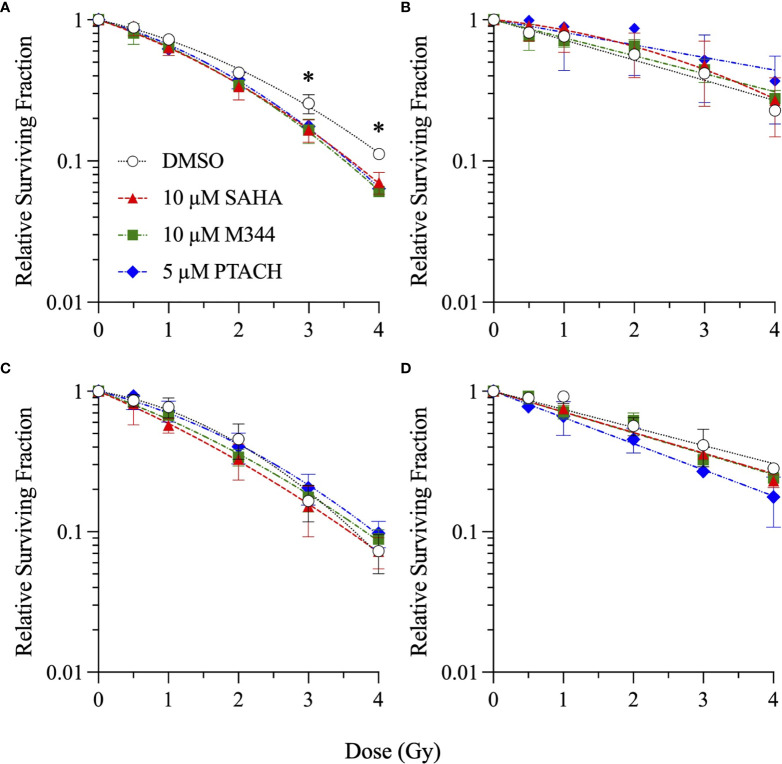
Clonogenic survival of G0/G1-phase normal NFF28 primary fibroblasts **(A)** and asynchronously growing A549 lung carcinoma **(B)**, U2OS osteosarcoma **(C)**, and U87MG malignant glioma cells **(D)** pretreated for 18 h with 10 µM SAHA, 10 µM M344, or 5 µM PTACH and exposed to 0.5–4 Gy 200 MeV protons in the Bragg plateau region (LET = 0.45 keV/µm). Data reported as mean ± SD; where error bars are not visible, they are smaller than the data point. Asterisks mark significant differences compared to DMSO controls at *p*-values of ≤0.05 (*) by one-way ANOVA.

**Figure 3:**
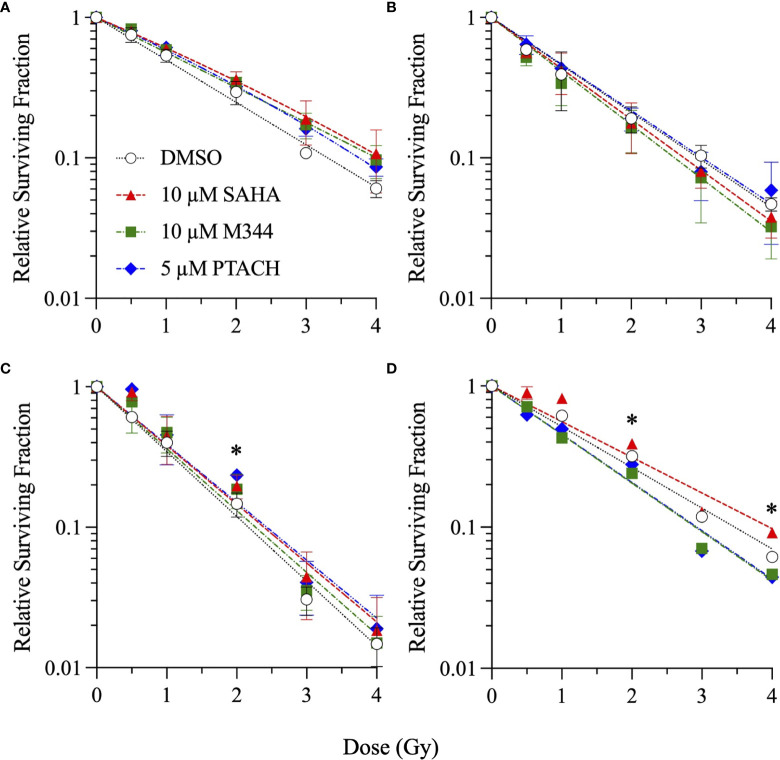
Clonogenic survival of G0/G1-phase normal NFF28 primary fibroblasts **(A)** and asynchronously growing A549 lung carcinoma **(B)**, U2OS osteosarcoma **(C)**, and U87MG malignant glioma cells **(D)** pretreated for 18 h with 10 µM SAHA, 10 µM M344, or 5 µM PTACH and exposed to 0.5–4 Gy 290 MeV/n C-12 ions in the Bragg plateau region (LET = 13.02 keV/µm). Data reported as mean ± SD; where error bars are not visible, they are smaller than the data point. Asterisks mark significant differences compared to DMSO controls at *p*-values of ≤0.05 (*) by one-way ANOVA.

**Figure 4:**
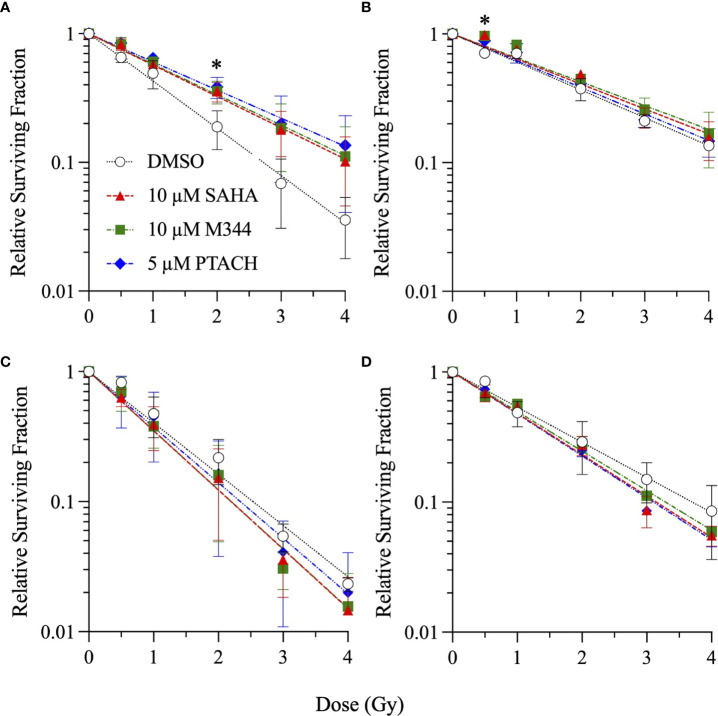
Clonogenic survival of G0/G1-phase normal NFF28 primary fibroblasts **(A)** and asynchronously growing A549 lung carcinoma **(B)**, U2OS osteosarcoma **(C)**, and U87MG malignant glioma cells **(D)** pretreated for 18 h with 10 µM SAHA, 10 µM M344, or 5 µM PTACH and exposed to 0.5–4 Gy 350 MeV/n O-16 ions in the Bragg plateau region (LET = 20.90 keV/µm). Data reported as mean ± SD; where error bars are not visible, they are smaller than the data point. Asterisks mark significant differences compared to DMSO controls at *p*-values of ≤0.05 (*) by one-way ANOVA.

Single-cell colony formation assays were next performed with 10 µM SAHA, 10 µM M344, and 5 µM PTACH concentrations to determine their ability to radiosensitize all four cell types to charged particle irradiation delivered at typical hadron RT energies. Unlike clinical SOBP irradiations employing the higher LET portion of the Bragg peak, these irradiations were conducted using the initial Bragg plateau region since a major focus of this study was modeling proximal (entrance) normal tissue effects of irradiated stromal fibroblasts. Survival curves are shown in **Figures 2** **–** **4** for low-passage G0/G1-phase NFF28 fibroblasts (**panel A**) and asynchronously growing A549 lung carcinoma (**B**), U2OS osteosarcoma (**C**), and U87MG malignant glioma cells (**D**) irradiated with 0.5–4 Gy of 200 MeV protons (LET = 0.45 keV/µm in H_2_O; **Figure 2**), 290 MeV/n carbon-12 ions (13.02 keV/µm; **Figure 3**), or 350 MeV/n oxygen-16 ions (20.90 keV/µm; **Figure 4**), respectively. All three HDACi were able to significantly radiosensitize NFF28 fibroblasts at higher proton doses of 3–4 Gy (*p* = 0.0001–0.0311; **Figure 2A**) and for 2 Gy of O-16 ions (*p* = 0.0179–0.0386; **Figure 4A**); however, no significant differences were noted for C-12 ion irradiations (**Figure 3A**). Significant HDACi-mediated radiosensitization was not observed for A549 cells exposed to any of the three charge particle types except for 50 cGy O-16 ion irradiations (*p* = 0.0193 for 10 µM SAHA and 0.025 for 10 µM M344; **Figure 4B**). The only significant difference observed for U2OS survival was for 5 µM PTACH-pretreated cells exposed to 2 Gy C-12 ions (*p* = 0.00422; **Figure 3C**). For U87MG survival, significant differences were only noted for 2 and 4 Gy C-12 ion irradiations (*p* = 0.0442 and 0.0296, respectively; **Figure 3D**).

**Figure 5:**
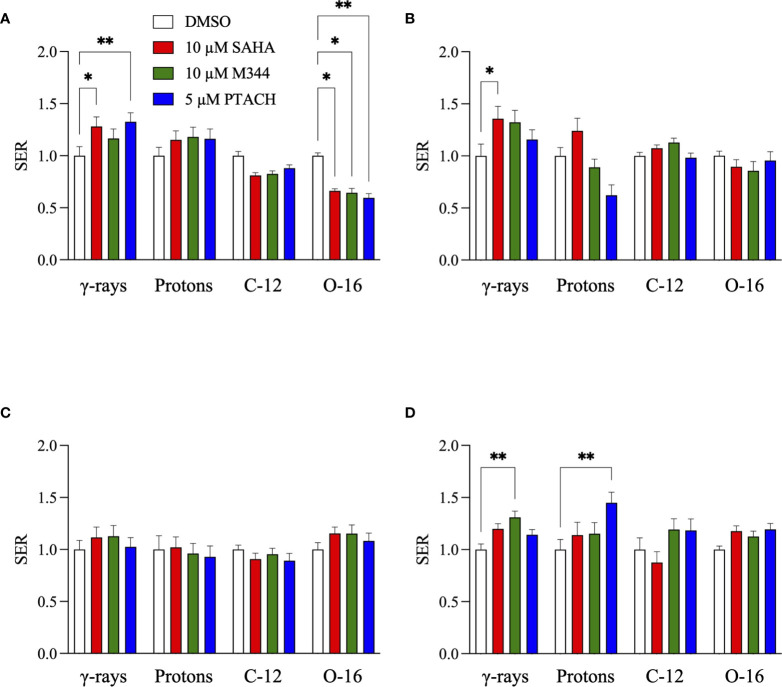
HDACi sensitizer enhancement ratio (SER) values ± SEM for G0/G1-phase normal NFF28 primary fibroblasts **(A)** and asynchronously growing A549 lung carcinoma **(B)**, U2OS osteosarcoma **(C)**, and U87MG malignant glioma cells **(D)** pretreated for 18 h with 10 µM SAHA, 10 µM M344, and 5 µM PTACH and irradiated with cesium-137 γ-rays, 200 MeV protons, 290 MeV/n C-12 ions, or 350 MeV/n O-16 ions [calculated using D_10_ survival values; asterisks mark significant differences at *p*-values of ≤0.05 (*) and ≤0.01 (**) by two-way ANOVA].

SER values for the three HDACi and four radiation qualities used in this study are shown in **Figure 5**. Significant levels of HDACi-mediated γ-ray radiosensitization were observed for NFF28 fibroblasts (*p* = 0.024 for 10 µM SAHA and 0.0075 for 5 µM PTACH by two-way ANOVA), A549 cells (*p* = 0.0123 for 10 µM SAHA), and U87MG cells (*p* = 0.0064 for 10 µM M344). Unlike the nearly uniform radiosensitization observed for γ-rays with all four cell types, pretreatment with the optimal HDACi γ-ray radiosensitizing concentrations yielded mixed results in terms of charged particle radiosensitization or radioprotection depending on the cell strain/line and ion species. For 200 MeV protons, levels of radiosensitization similar to γ-rays were observed for quiescent NFF28 fibroblasts and U87MG cells only (**Figures 2A, D**; *p* =0.0096 for U87MG cells pretreated with 5 µM PTACH). For A549 lung carcinoma cells (**Figure 2B**), pretreatment with 10 µM SAHA radiosensitized cells to protons; however, pretreatment with 10 µM M344 and 5 µM PTACH conversely provided radioprotection. For U2OS osteosarcoma cells (**Figure 2C**), only pretreatment with 10 µM SAHA showed slight radiosensitization for protons, while the other two HDACi provided mild radioprotection. Results for the intermediate LET C-12 and O-16 ion irradiations were even more surprising, with all three HDACi providing radioprotection for quiescent G0/G1-phase NFF28 fibroblasts (significantly so for O-16 ions, *p* = 0.0054–0.023; **Figures 3A**, **4A**, **5A**). For the three tumor cell lines, patterns of HDACi-mediated radiosensitization and radioprotection varied for C-12 and O-16 ions. A549 cells showed mild HDACi-mediated radiosensitization for C-12 ions and radioprotection for O-16 ions (**Figures 3B**, **4B**). U2OS cells showed the reverse pattern whereby the inhibitors provided mild radioprotection for C-12 ions and radiosensitization for O-16 ions (**Figures 3C**, **4C**). For U87MG cells, HDACi pretreatment generally resulted in radiosensitization for both ions (**Figures 3D**, **4D**), although pretreatment with 10 µM SAHA resulted in C-12 ion radioprotection.

**Figure 6:**
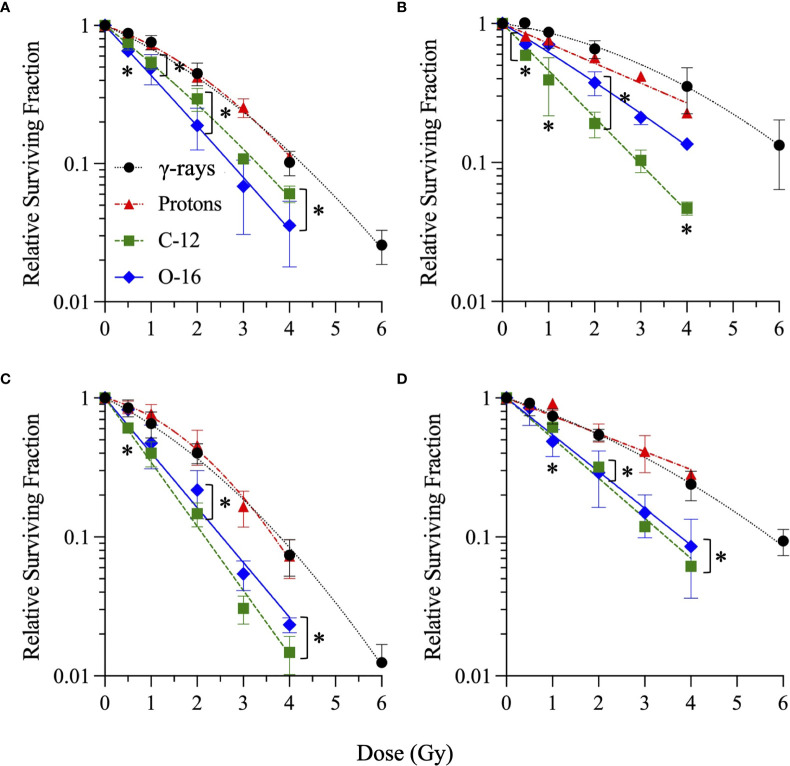
Clonogenic survival of DMSO-treated (vehicle control) G0/G1-phase normal NFF28 primary fibroblasts **(A)** and asynchronously growing A549 lung carcinoma **(B)**, U2OS osteosarcoma **(C)**, and U87MG malignant glioma cells **(D)** irradiated with cesium-137 γ-rays, 200 MeV protons, 290 MeV/n C-12 ions, or 350 MeV/n O-16 ions. Data reported as mean ± SD; where error bars are not visible, they are smaller than the data point. A LET-dependent increase in cell killing is observed for NFF28 fibroblasts; however 290 MeV/n C-12 ions (LET = 13.02 keV/µm) were more effective than 350 MeV/n O-16 ions (LET = 20.90 keV/µm) for cell killing in all three tumor cell lines. Asterisks mark significant differences compared to cesium-137 γ-rays at *p*-values of ≤0.05 (*) by one-way ANOVA.

In order to compare responses to the different radiation types alone (without HDACi treatments), survival curves for DMSO-treated vehicle controls are shown in **Figure 6**. Per **Figure 6A**, there is a direct correlation of cell killing with particle LET in G0/G1-phase NFF28 fibroblasts, with exposures to cesium-137 γ-rays and 200 MeV Bragg plateau protons yielding approximately equivalent survival. Both C-12 and O-16 ion irradiations resulted in significantly increased NFF28 cell killing compared to γ-rays (*p* = 0.015–0.0268 for C-12 ions and 0.0008–0067 for O-16 ions by one-way ANOVA). Significantly increased tumor cell killing following C-12 and O-16 ion irradiations was also observed for A549 cells (*p* = 0.0002–0.0385 for C-12 ion and 0.0011–0.0146 for O-16 ions), U2OS cells (*p* = 0.0032–0.0137 for C-12 ions and 0.0084–0.0429 for O-16 ions), and U87MG cells (*p* = 0.0104–0.0303 for C-12 ions and 0.0179–0.0438 for O-16 ions). Interestingly, in all three tumor lines, cell killing was greater for 290 MeV/n Bragg plateau C-12 ions (LET = 13.02 keV/µm) compared to higher LET 350 MeV/n Bragg plateau O-16 ions (LET = 20.90 keV/µm; **Figures 6B–D**). For proton irradiations, only A549 cells showed increased cell killing compared to γ-rays (*p* = 0.0094 at 50 cGy).

**Figure 7:**
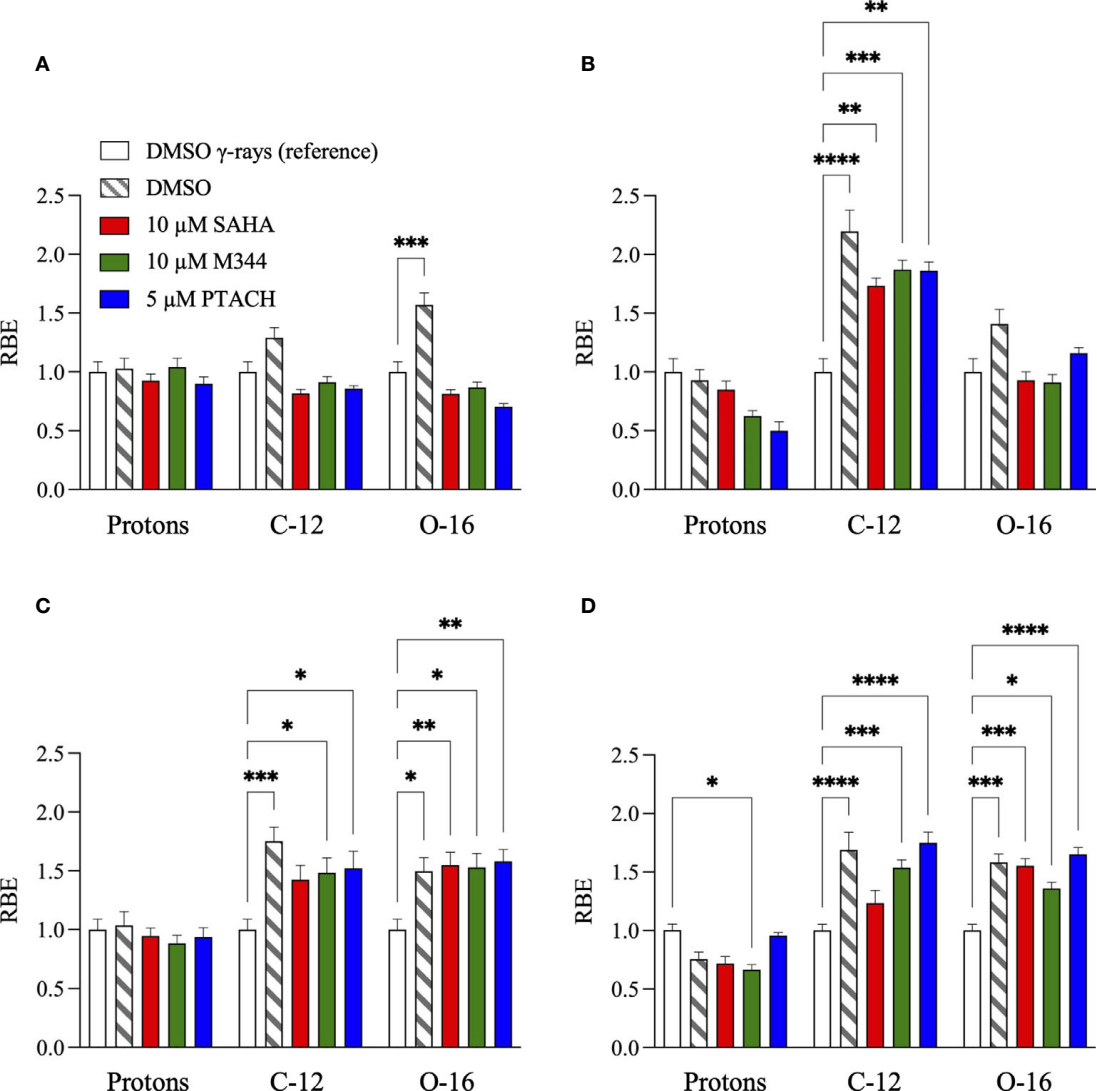
Relative biological effectiveness (RBE) values ± SEM for G0/G1-phase normal NFF28 primary fibroblasts **(A)** and asynchronously growing A549 lung carcinoma **(B)**, U2OS osteosarcoma **(C)**, and U87MG malignant glioma cells **(D)** pretreated for 18 h with 10 µM SAHA, 10 µM M344, and 5 µM PTACH and irradiated 200 MeV protons, 290 MeV/n C-12 ions, or 350 MeV/n O-16 ions compared to respective DMSO control or HDACi-pretreated cultures exposed to reference cesium-137 γ-rays [calculated using D_10_ survival values; asterisks mark significant differences at *p*-values of ≤0.05 (*), ≤0.01 (**), ≤0.001 (***), and ≤10^−4^ (****) by two-way ANOVA].

To summarize the charged particle clonogenic survival results from DMSO and HDACi-treated cells in **Figures 2**–**4**, RBE values for the three radiation types compared to reference cesium-137 γ-rays (**Figure 1** and **Supplementary Figures 2**, **3**) are reported in **Figure 7** with RBE values for HDACi-treated cells calculated from corresponding γ-ray survival D_10_ values of cells pretreated with the identical HDACi concentrations. In this case, RBE values <1 would imply that the particular combination of charged particle and HDACi was less effective for cell killing than the corresponding γ-ray/HDACi combination. For protons, HDACi pretreatment provided no added advantage compared to exposing pretreated cultures to γ-rays and often was less effective (significantly so for U87MG cells pretreated with 10 µM M344, *p* = 0.0365). For C-12 ions, DMSO and HDACi-pretreated cells yielded significantly higher RBE values by ANOVA analyses in all three tumor cell lines (*p* = <0.0001–0.0057 for A549 cells, 0.0002–0.0241 for U2OS cells, and <0.0001–0.0003 for U87MG cells), and the HDACi treatments nearly uniformly resulted in tumor cell radioprotection compared to vehicle controls. Significantly increased cell killing was observed in NFF28 fibroblasts following O-16 ion irradiation (*p* = 0.0002), but this effect was lost in the HDACi-pretreated cultures. Significantly increased U2OS and U87MG tumor cell killing was also observed for O-16 ion irradiations (*p* = 0.0048–0.0197 and <0.0001–0.0225, respectively), with little added effect seen following HDACi pretreatments. In NFF28 fibroblasts and A549 cells, HDACi pretreatments resulted in radioprotection for O-16 ion exposures. **Supplementary Table 2** summarizes the various D_10_, HDACi SER, and charged particle RBE values for the four cell types.

### DSB-Associated Foci Assays

**Figure 8:**
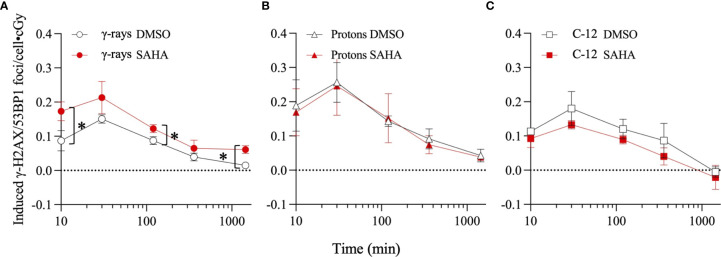
Dose-corrected γ-H2AX pS139/53BP1 foci levels in quiescent G0/G1-phase normal NFF28 primary fibroblasts pretreated for 18 h with 0.1% DMSO (vehicle control) or 10 µM SAHA measured by immunocytochemistry/image analysis in samples fixed 10 min to 24 h post-irradiation following low dose (5–25 cGy) exposures of cesium-137 γ-rays **(A)**, 200 MeV protons **(B)**, and 290 MeV/n C-12 ions **(C)**. Peak foci formation occurs at 30 min post-IR for all three radiation qualities. Data reported as IR-induced means ± SD, background-subtracted for foci levels measured in sham-irradiated cultures (indicated by dashed line). Asterisks mark significant differences compared to DMSO controls at *p*-values of ≤0.05 (*) by two-tailed Student’s *t*-tests.

Results of γ-H2AX/53BP1 foci analyses conducted in quiescent G0/G1-phase cultures of NFF28 fibroblasts pretreated for 18 h with 10 µM SAHA and subsequently irradiated with 5–25 cGy doses of cesium-137 γ-rays, 200 MeV protons, and 290 MeV/n C-12 ions are shown in **Figures 8A–C**, respectively, reported as numbers of mean (± SEM) IR-induced foci per unit dose. Multiple post-translational modifications of histone H2AX and 53BP1 occur after the initial recognition of IR-induced DSBs, including the phosphorylation of H2AX on serine 139 (γ-H2AX pS139) by the DDR kinases ATM and DNA-PK (54–56) and 53BP1 recruitment and binding to post-translationally modified histones (57–61). The formation and colocalization of these cytogenetically visible DSB-associated foci permit their more efficient detection and enumeration at low IR doses (50, 62, 63). Peak DSB-associated foci induction was observed 30 min post-irradiation for all three radiation types, followed by similar rates of NHEJ-mediated DSB repair and foci resolution to at or near background levels by 24 h, similar to the low dose responses of other normal primary fibroblast strains reported in (50). Mean background levels of foci measured in sham-irradiated cultures were 1.02 foci/cell for DMSO-pretreated cultures and 0.99 foci/cell for 10 µM SAHA-pretreated cultures. The greatest amount of radiosensitization, measured as ~1.4-fold increased levels of peak induced foci formation, was observed following cesium-137 γ-ray irradiation (**Figure 8A**). Significant differences in γ-ray-induced foci levels between DMSO vehicle and 10 µM SAHA pretreatments were observed 10 min post-IR (*p* = 0.0216), as well as at the 6 and 24 h timepoints (*p* = 0.0208 and 0.0144, respectively). Low-dose proton-induced foci levels were similar in control and SAHA-pretreated cultures (**Figure 8B**), and foci levels were reduced ~1.4-fold in 10 µM SAHA-pretreated cells following low dose C-12 ion irradiation (**Figure 8C**). These patterns reflect the results of the clonogenic assay results whereby the greatest degree of low dose radiosensitization is observed for cesium-137 γ-rays, followed by similar survival of control and SAHA-pretreated cells following low-dose proton irradiation, and radioprotection observed for C-12 ion-irradiated NFF28 fibroblasts pretreated with SAHA.

### NFF28 Asynchronous Cell Survival and Transformation Assays

**Figure 9:**
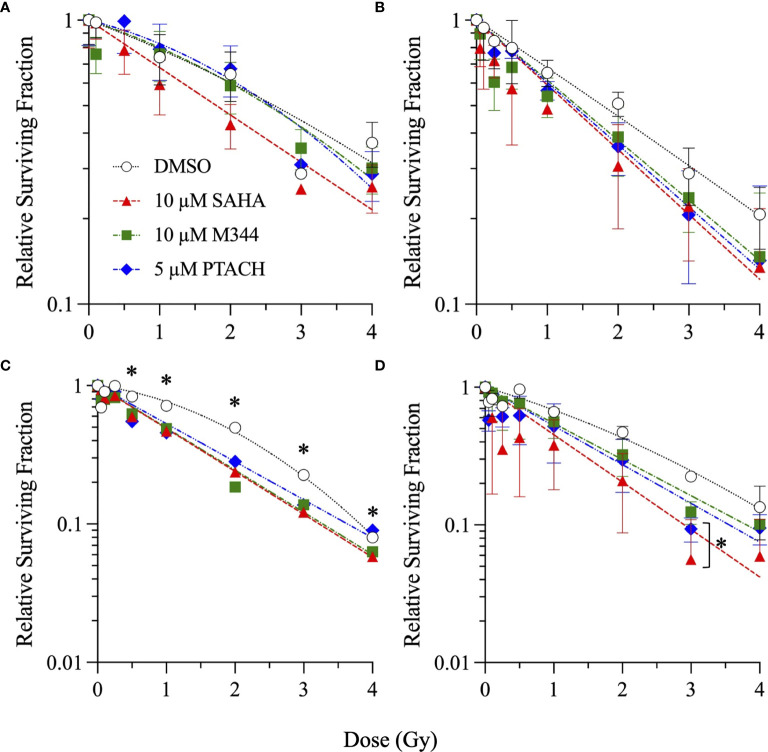
Clonogenic survival of asynchronously growing normal NFF28 primary fibroblasts pretreated for 18 h with 10 µM SAHA, 10 µM M344, and 5 µM PTACH and irradiated cesium-137 γ-rays **(A)**, 200 MeV protons **(B)**, 290 MeV/n C-12 ions **(C)**, or 350 MeV/n O-16 ions **(D)**. Data reported as mean ± SD; where error bars are not visible, they are smaller than the data point. Unlike the radioprotection observed following HDACi pretreatment in G0/G1-phase cultures irradiated with C-12 or O-16 ions, 18 h HDACi pretreatment of asynchronously growing NFF28 fibroblast cultures resulted in radiosensitization. Asterisks mark significant differences compared to DMSO controls at *p*-values of ≤0.05 (*) by one-way ANOVA.

**Figure 10:**
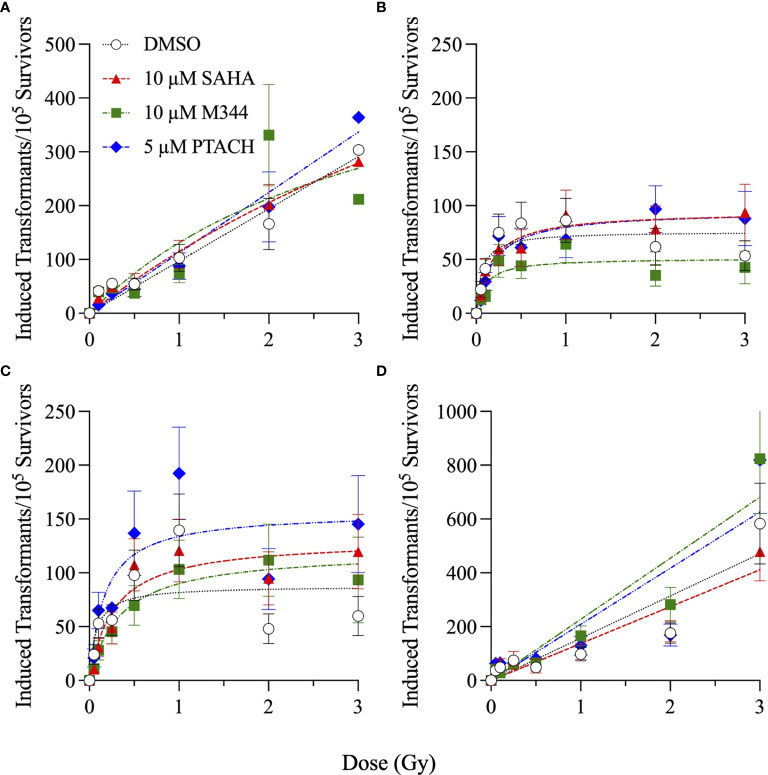
Cellular transformation of asynchronously growing normal NFF28 primary fibroblasts pretreated for 18 h with 10 µM SAHA, 10 µM M344, and 5 µM PTACH and irradiated cesium-137 γ-rays **(A)**, 200 MeV protons **(B)**, 290 MeV/n C-12 ions **(C)**, or 350 MeV/n O-16 ions **(D)** measured as the acquisition of anchorage-independent colony formation in soft agar per 10^5^ surviving cells. Data reported as mean ± SEM; where error bars are not visible, they are smaller than the data point. A hyperlinear response is observed for low-dose exposures with transformation frequencies peaking at 1 Gy and then declining at higher doses following proton and C-12 ion irradiation.

Finally, the clonogenic survival of asynchronously growing NFF28 fibroblasts and frequencies of *in vitro* cellular transformation, measured as the acquisition of anchorage-independent (A-I) growth in soft agar, following HDACi pretreatment and irradiation with cesium-137 γ-rays, 200 MeV protons, 290 MeV/n C-12 ions, and 350 MeV/n O-16 ions are shown in **Figures 9**, **10**, respectively. These assays were conducted using protocols devised by Dr. Betsy Sutherland and colleagues that utilize asynchronously growing log-phase human fibroblast cultures rather than quiescent G0/G1-phase cultures. Following a 7-day culture recovery period to allow clearance of dead cells, surviving irradiated NFF28 fibroblasts were subcultured, plated at low density in soft agar, and incubated for a 20-day colony formation period after which the dishes were rinsed and evaluated for A-I colonies. In contrast to the G0/G1-phase survival assay results reported in previous figures whereby HDACi pretreatment was radiosensitizing for γ-ray and proton irradiations but radioprotective for C-12 and O-16 ion irradiations, 18 h HDACi pretreatment of asynchronously growing log-phase NFF28 cultures resulted in sensitization for *all* radiation types as shown in **Figure 9** (suggesting that the higher proportion of S and G2-phase cells in these cultures are more sensitive to HDAC inhibition and subsequent charged particle irradiation than G0/G1-phase cells). Of the three HDACi tested, pretreatments with 10 µM SAHA consistently yielded the best radiosensitization; however, neither 10 µM M344 nor 5 µM PTACH was effective at radiosensitizing asynchronously growing NFF28 cells to γ-rays. Significant differences in clonogenic survival between HDACi and DMSO vehicle-pretreated cells were observed primarily for C-12 ions (**Figure 9C**; *p*-values ranging from ≤0.0001 to 0.0334 by one-way ANOVA), while only SAHA and PTACH were able to significantly radiosensitize cells to higher doses of O-16 ions (*p* = 0.0127 and 0.0304 at 3 Gy, respectively).

Frequencies of IR-induced NFF28 cellular transformation, measured as the yields of A-I colonies per 10^5^ surviving cells, are plotted in **Figure 10**. The lowest overall induction occurred following 200 MeV proton irradiation (~0.74-fold that of cesium-137 γ-rays) with C-12 and O-16 ion irradiations producing ~1.7-fold and ~3.2-fold higher maximal yields of transformants per unit dose compared to cesium-137 γ-rays, respectively. All four radiation qualities demonstrate a rapid increase in transformation induction for doses ≤50 cGy (significantly so for γ-ray and proton exposures), a plateauing at 1 Gy, and in the case of protons and C-12 ions, decreasing yields at higher doses. Both cesium-137 γ-rays and O-16 ions also showed a general plateau at higher doses up to 2 Gy; however, yields of transformants were further increased at 3 Gy for both radiation types. Yields of γ-ray and proton-induced transformants per unit dose measured at low doses (≤25 cGy) were ~1.8 and 2.8-fold higher, respectively, than yields measured at higher doses (>50 cGy), with ratios of ~1.3 and ~1 calculated for C-12 and O-16 ions, respectively. HDACi pretreatment in the majority of cases resulted in decreased yields of transformants at low doses (≤25 cGy) and increased yields of transformants for all radiation types at higher doses (>50 cGy). The only exceptions were M344 pretreatment followed by proton irradiation and SAHA pretreatment followed by O-16 ion irradiation. Although per **Figure 9**, HDACi pretreatments were radiosensitizing for asynchronously NFF28 fibroblast survival, the fact that yields of transformants are weighted and reported per number of *surviving* cells implies that HDACi treatments generally increased both γ-ray and charged particle-induced normal fibroblast transformation overall (although the differences were not statistically significant).

**Figure 11:**
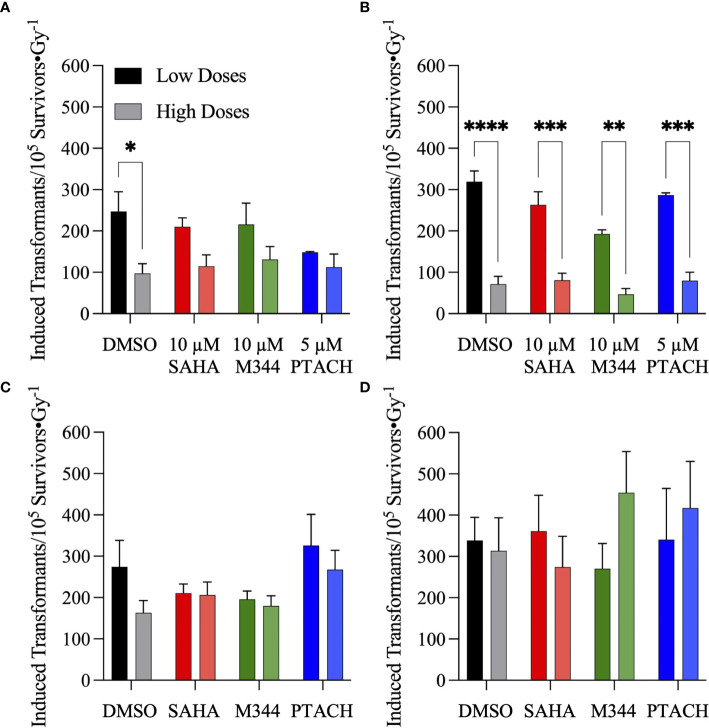
Frequencies of normal NFF28 primary fibroblast anchorage-independent (A–I) colony formation in soft agar per unit dose ± SEM at low (≤25 cGy) and higher doses (>50 cGy) of cesium-137 γ-rays **(A)**, 200 MeV protons **(B)**, 290 MeV/n C-12 ions **(C)** and 350 MeV/n O-16 ions **(D)**. IR-induced yields of transformants are background-corrected for levels of transformants measured in DMSO and HDACi-treated/sham-irradiated cultures (~6.1–7.2). Low dose transformation frequencies are calculated from the slope of induction from 5 to 25 cGy and the higher dose transformation frequencies obtained from the mean transformation frequencies at 1 Gy, the dose where peak (maximal) transformation occurs for both protons and C-12 ions. Both cesium-137 γ-rays and protons show a pronounced hyper-radiosensitivity for NFF28 fibroblast *in vitro* transformation at low doses. Asterisks mark significant differences at *p*-values of ≤0.05 (*), ≤0.01 (**), ≤0.001 (***), and ≤10^−4^ (****) by two-way ANOVA.

Given the steep increase in yield of transformants at low doses, the frequencies of IR-induced NFF28 fibroblast transformation per unit dose at low doses (≤25 cGy) and higher doses (>50 cGy) following SAHA, M344, or PTACH pretreatment are comparatively plotted in **Figure 11**. Low dose transformation frequencies were calculated as the slope of induction from 5–25 cGy, and the higher dose transformation frequencies were obtained from the mean transformation frequencies at 1 Gy, the dose where peak (maximal) transformation occurs for both 200 MeV protons and 290 MeV/n C-12 ions (**Figures 10B, C**). As per **Figures 11A, B**, both cesium-137 γ-rays and protons showed significantly higher yields of transformants per unit dose at low doses compared to higher doses (*p* = 0.0477 and <0.0001–0.003, respectively, by two-way ANOVA), with HDACi pretreatment reducing the yields of low dose transformants for both radiation types compared to DMSO-treated controls. This pattern of low dose transformation sparing is likewise observed for 18 h PTACH pretreatment preceding C-12 ion irradiation and SAHA pretreatment prior to O-16 ion irradiation. Pretreatment with either SAHA or M344 followed by C-12 ions resulted in equivalent yields of transformants at both low and high doses, and pretreatment with M344 or PTACH prior to O-16 ion irradiation increased transformation at higher doses. Low-dose hyper-radiosensitivity for NFF28 fibroblast transformation was generally not observed for C-12 or O-16 ions, suggesting it may be a phenomenon particular to low LET radiations. Results of the clonogenic survival and transformation experiments conducted with asynchronously growing NFF28 fibroblasts are summarized in **Supplementary Tables 3**, **4** (D_10_ values and yields of transformants per unit dose, respectively, along with associated HDACi SER and charged particle RBE values).

## Discussion

In light of previous reports of promising levels of SAHA and other HDACi-induced X- and γ-ray radiosensitization, we sought to determine if these agents would be likewise useful for radiosensitizing tumor cells to charge particle-based radiotherapy modalities (without likewise doing so to surrounding normal tissues) using normal diploid human fibroblasts and three tumor cell lines as *in vitro* models. Few reports exist on the utility of using HDACi as adjuvants for proton or carbon ion radiotherapies, with available studies generally showing increased tumor cell killing *in vitro via* clonogenic survival assays and increased levels of IR-induced DSBs measured by DSB-associated foci assays, often with delayed foci repair kinetics in HDACi-treated cells compared to vehicle-treated controls. In this study, we validated that the clinically approved HDACi SAHA and two related hydroxamate analogues M344 and PTACH were effective for radiosensitizing normal human fibroblasts and tumor cells to cesium-137 γ-rays and 200 MeV protons (**Figures 1**, **2**). However, the three HDACi tested provided comparatively less radiosensitization (and in some case radioprotection) to pretreated tumor cells prior to C-12 or O-16 ion irradiation (**Figures 3**, **4**). Effects of HDACi pretreatment in NFF28 fibroblasts depended on whether cultures were quiescent or cycling, with radioprotection afforded to stationary G0/G1-phase cultures exposed to C-12 and O-16 ions. HDACi pretreatment radiosensitized log-phase NFF28 cells to these ions (**Figure 9**) and generally increased the frequencies of IR-induced transformation following exposure to all four radiation types (**Figure 10**). These studies therefore provide mixed results on the utility of HDACi for increasing the effectiveness of light ion-based hadron radiotherapies.

The clonogenic survival of A549 lung carcinoma and diploid normal fibroblasts reported herein are very similar to those reported in (38) who used 24 h pretreatments of 0.2 or 2 µM SAHA concentrations prior to irradiation (compared to 18 h pretreatments in our study). We both report cesium-137 γ-ray D_10_ values of 6.5 Gy for control (DMSO-treated) A549 cells and very similar D_10_ values of 4.31 and 4.27 Gy for two low-passage G0/G1-phase primary fibroblast strains (AG01522 and NFF28, respectively; **Supplementary Table 1**). From their report, pretreatment with 2 µM SAHA results in increased γ-ray radiosensitization in both cell types (D_10_ values of 4.67 Gy for A549 cells and 4.21 Gy for AG01522 fibroblasts) similar to the 1 and 5 µM SAHA pretreatment D_10_ values of 4.27–4.78 Gy for A549 cells and 3.43–4.34 Gy for NFF28 fibroblasts reported in **Supplementary Table 1**. For survival following 200 MeV SOBP proton irradiations (LET = 2.2 keV/µm), this group also documented that SAHA pretreatment radiosensitized both cell types. However, per **Figure 2B**, pretreatment of A549 cells with SAHA and the other two HDACi used in our studies resulted in radioprotection following proton irradiation (**Figure 2** and **Supplementary Table 2**). This group likewise observed radiosensitization in SAHA-pretreated A549 cells exposed to 290 MeV/n SOBP C-12 ions (LET = 55 keV/µm), as we did in **Figure 3B**, but did not observe any differences in the survival of DMSO *versus* SAHA-treated AG01522 cells. Since we used a 5-fold higher SAHA concentration, it is possible that the 2 µM concentration used in their experiments may not have been sufficient to provide the radioprotection we observed for NFF28 cells exposed to C-12 or O-16 ions (**Figures 3A**, **4A**). It should again also be noted that our experiments employed 200 MeV proton and 290 MeV/n C-12 ion irradiations delivered in the lower LET Bragg plateau/entrance region (LET values of 0.45 and 13 keV/µm, respectively) to mimic normal tissue entrance exposures. As such, we report slightly higher corresponding D_10_ values for experiments presented herein—though the patterns and relative degree of SAHA-mediated radiosensitization or radioprotection are similar (**Supplementary Table 2**). Levels of C-12 ion-induced cell killing in DMSO-treated NFF28 fibroblasts and U87MG cells in this report are consistent with entrance region (13 keV/µm) C-12 ion exposures of NB1RBG fibroblasts reported in (64) and U87MG cells in (65) delivered at the HIMAC facility in Chiba, Japan.

As seen in **Figures 3**, **4**, HDACi-mediated C-12 and O-16 ion radiosensitization was cell line/strain- and HDACi-specific. Along with NFF28 fibroblasts (**Figure 3A**), HDACi pretreatment of U2OS osteosarcoma cells was also radioprotective for C-12 ion exposures (**Figure 3C**), as was PTACH pretreatment in A549 cells and SAHA pretreatment in U87MG cells (**Figures 3B, D**). Conversely, M344 and SAHA pretreatment radiosensitized A549 cells and M344 and PTACH radiosensitized U87MG cells to C-12 ion irradiation. For O-16 ion irradiations, pretreatment of quiescent G0/G1-phase NFF28 fibroblasts with each of the three HDACi was strongly radioprotective (**Figure 4A**) and likewise conferred mild radioprotection to A549 cells (**Figure 4B**). Only HDACi pretreatments of U2OS and U87MG cells were effective in moderately radiosensitizing them to subsequent O-16 ion exposures (**Figures 4C, D**). Another interesting result is seen in **Figure 6** in which cell survival is plotted for DMSO vehicle-treated controls only. While the canonical pattern of increased LET-dependent cell killing is seen for NFF28 fibroblasts, whereby 350 MeV/n O-16 ion (20.9 keV/µm) exposures are more effective at cell killing than 290 MeV/n C-12 ion (13 keV/µm) exposures (**Figure 6A**), for all three tumor lines, the lower LET C-12 ions are more effective for cell killing compared to O-16 ions (**Figures 6B–D**). It remains to be determined if this would be repeated using higher LET SOBP C-12 and O-16 ion irradiations.

Unlike the report by (38) and other groups that have also examined the effects of SAHA pretreatment on post-irradiation γ-H2AX and 53BP1 foci induction and repair kinetics, we did not identify any significant effects of inhibitor pretreatment on the *rates* of DSB repair and accompanying foci resolution, rather only observed differences in the overall foci induction *levels* post-IR. It should also be noted that many of the studies that describe HDACi-induced delayed γ-H2AX foci repair kinetics report results from human tumor cell lines (36, 66). We observed increased colocalized γ-H2AX pS139/53BP1 foci induction in SAHA-pretreated NFF28 fibroblasts following cesium-137 γ-irradiation, which, unlike DMSO-treated vehicle controls, did not return to baseline levels by 24 h (**Figure 8A**). Similar results were documented in (67) demonstrating radiosensitization for γ-H2AX foci induction in G0/G1-phase HSF1 fibroblasts pretreated for 12 h with 10 µM SAHA prior to irradiation with 0.5 Gy of 90 kV X-rays. This significant increase in γ-ray-induced foci levels following 10 µM SAHA pretreatment shown in **Figure 8A** coincides with the increased radiosensitivity for clonogenic survival in **Figure 1A**. No differences in γ-H2AX/53BP1 foci induction levels were noted in DMSO *versus* SAHA-pretreated NFF28 cells exposed to 200 MeV protons in this study (**Figure 8B**), similar to results reported in (38), and levels of residual foci at 24 h remained above background in these cultures. Similarly, little difference in clonogenic survival was seen between DMSO control and SAHA-treated NFF28 fibroblasts irradiated with low-dose 200 MeV protons per **Figure 2A**. However, unlike the Gerelchuluun et al. report, we identified SAHA pretreatment as radioprotective (rather than radiosensitizing) for C-12 ion-induced peak DSB-associated foci induction with *lower* levels of DSB-associated foci induced per unit dose in irradiated cultures, as well as levels of IR-induced foci falling below background levels by 24 h (**Figure 8C**). The lower induction of foci following C-12 ion irradiation coincides with our finding of increased survival (radioprotection) in C-12 ion-irradiated G0/G1-phase NFF28 fibroblasts pretreated with 10 µM SAHA in **Figure 3A** [a finding likewise reported for confluent AG01522 fibroblast survival in (38)]. Lower levels of γ-H2AX expression measured by flow cytometry along with increased cell survival were also observed in 1 µM SAHA-treated hFOB 1.19 osteoblast cells irradiated with C-12 ions at the HIT facility in Heidelberg, Germany, as reported by (35). Thus, for the three radiation types used in **Figure 8**, effects of 10 µM SAHA pretreatment on the relative induction of DSB-associated foci directly matched the respective patterns of radiosensitization or radioprotection observed for NFF28 clonogenic survival (**Figures 1**–**3**).

Irradiation with 200 MeV protons produced the greatest number of induced colocalized γ-H2AX/53BP1 foci per unit dose (0.26 foci/cGy), followed by 290 MeV/n C-12 ions (0.18 foci/cGy) and cesium-137 γ-rays (0.15 foci/cGy; **Figure 8**). A recent study by (68) likewise documented lower γ-H2AX and 53BP1 foci induction per Gy in human TIG-3-20 fibroblasts irradiated with 190 MeV C-12 ions compared to 20 MeV protons and 63-MeV He-4 ions, noting that it is likely multiple DSBs would be contained with a single C-12 ion-induced focus given the higher density of ionizations along individual particle trajectories of these higher LET-charged particles. Assuming a mean G0/G1-phase human fibroblast cell nucleus surface area of ~200 µm^2^ (69, 70), exposure to entrance region 290 MeV C-12 ions (LET = 13.02 keV/µm) in our experiments results in ~1 C-12 ion traversal/nucleus per cGy compared to ~29.2 entrance region 200 MeV proton (LET = 0.45 keV/µm) traversals/nucleus per cGy and ~14.5 cesium-137 γ-ray-induced photoelectron [LET = 0.91 keV/µm at full buildup (71),] traversals/nucleus per cGy. This equates to ~0.009 induced foci/proton, 0.01 induced foci/photoelectron, and ~0.18 induced foci/C-12 ion: relative ratios of ~20:1 for 290 MeV/n C-12 ions and 1.15:1 for γ-rays compared to 200 MeV protons in this case. Since the relative ratios of their respective LET values are ~33.4 for 290 MeV/n C-12 ions and ~2.3 for cesium-137 γ-rays compared to 200 MeV protons, respectively, there is a correlation between particle LET and DSB-associated foci induction levels when reported per particle fluence (rather than per unit dose).

To our knowledge, this is the first study to report frequencies of IR-induced normal cell transformation *in vitro* following HDACi pretreatment. Cellular transformation assays assessing anchorage-independent growth in soft agar of irradiated primary human fibroblasts or CGL1 HeLa/fibroblast hybrid cells (72) have been employed in radiobiology for decades as a surrogate *in vitro* model capable of recapitulating some of the essential features of *in vivo* human carcinogenesis (73). The frequencies of NFF28 fibroblast transformation following cesium-137 γ-rays, 200 MeV protons, 290 MeV/n C-12 ions, and 350 MeV/n O-16 ions we measured in this study (**Figures 10**, **11**) are similar to those reported previously by the Sutherland group for low-passage NFF28 fibroblasts exposed to other proton energies, 250 kVp X-rays and intermediate to high LET Si-28, Ti-48, and Fe-56 ions (45–48). Overall, the HDACi pretreatments did not significantly affect NFF28 fibroblast transformation induction for any of the four radiation types tested (**Figure 10**), although 10 µM SAHA pretreatment resulted in increased proton and C-12 ion-induced transformation at higher doses. The hyperlinear increase in cellular transformation we observed at low doses (≤25 cGy) of protons and C-12 ions documented in these figures is concerning when considering exposures to normal tissues in irradiated entrance regions prior to the targeted tumor volume that may accrue over the course of a typical radiotherapy treatment regimen. It is also relevant for the radiation protection of astronauts exposed to the complex, chronic mixed-field space radiation environment during long-duration missions to the Moon and Mars, and potentially high doses they may receive during solar particle events (SPE) (74, 75). However, at higher (clinically relevant) doses used in this study, protons do appear to be less capable of inducing normal fibroblast transformation than cesium-137 γ-rays. This is reflected in a recent pooled cohort analysis of secondary cancer induction following clinical megavoltage photon- and electron-based IMRT or proton-based PBRT that shows incidence is approximately threefold higher for IMRT compared to PBRT (76). While our results show higher transformation following C-12 or O-16 ions compared to γ-rays, there are no clinical reports demonstrating C-12 ion-induced second cancer induction in CIRT patients given the low numbers of patients treated to date with this modality and long latency periods associated with solid cancer development (4, 77). Risk projections for CIRT-induced second cancer induction suggest comparable overall incidence compared to PBRT (78).

Overall, unlike the promising levels of HDACi-mediated radiosensitization observed for cesium-137 γ-rays (**Figures 1**, **5**), HDACi pretreatments resulted in generally more modest levels of radiosensitization for protons (and radioprotection in the case of A549 and U2OS cells pretreated with either M344 or PTACH; **Figures 2**, **5**). For C-12 ion irradiations, HDACi pretreatments had more minimal effects on the post-irradiation survival of the three tumor cell lines tested (**Figure 3**), while slight radiosensitization was observed for U2OS and U87MG cells exposed to O-16 ions (**Figure 4**). Survival assays using NFF28 fibroblasts showed the HDACi were effective for radiosensitizing both quiescent G0/G1-phase and log-phase cultures to γ-rays and protons, but were radioprotective when quiescent cultures were irradiated with C-12 and O-16 ions (**Figures 1**, **2**, **9**). This radioprotective effect was recapitulated in the DSB-associated foci assay results shown in **Figure 8** whereby lower levels of foci were observed in 10 µM SAHA-pretreated quiescent NFF28 cultures. Finally, while HDACi pretreatments were radiosensitizing to cycling NFF28 cells exposed to all three charged particle types, the increased yields of transformants measured in HDACi-treated cultures at higher doses in many cases (**Figures 10**, **11**) suggest they may possibly be associated with higher incidence of secondary cancer induction if utilized as PBRT or CIRT adjuvants.

## Data Availability Statement

The original contributions presented in the study are included in the article/**Supplementary Material**. Further inquiries can be directed to the corresponding author.

## Author Contributions

PW, DK, and PB conceived and designed the experiments. AJ, KS, AH, PB, JJ, DK, and PW performed the experiments. PW, AJ, DK, and PB summarized and analyzed the data. PW, AJ, DK, and PB wrote the paper. All authors contributed to the article and approved the submitted version.

## Conflict of Interest

The authors declare that the research was conducted in the absence of any commercial or financial relationships that could be construed as a potential conflict of interest.

## Publisher’s Note

All claims expressed in this article are solely those of the authors and do not necessarily represent those of their affiliated organizations, or those of the publisher, the editors and the reviewers. Any product that may be evaluated in this article, or claim that may be made by its manufacturer, is not guaranteed or endorsed by the publisher.

## Supplementary Material

Supplementary Figure 1 Sham-irradiated plating efficiencies (PE) ± SEM for G0/G1-phase normal NFF28 primary fibroblasts **(A)** and asynchronously growing A549 lung carcinoma **(B)**, U2OS osteosarcoma **(C)**, and U87MG malignant glioma cells **(D)** pretreated for 18 h with 0.1% DMSO (vehicle control) or 1–20 µM concentrations of SAHA, M344, and PTACH. Asterisks mark significant differences at *p*-values of ≤0.05 (*), ≤0.01 (**), ≤0.001 (***), and ≤10^−4^ (****).

Supplementary Figure 2 Clonogenic survival of G0/G1-phase normal NFF28 primary fibroblasts **(A)** and asynchronously growing A549 lung carcinoma **(B)**, U2OS osteosarcoma **(C)**, and U87MG malignant glioma cells **(D)** pretreated for 18 h with 1–20 µM concentrations of M344 and exposed to 0.5–6 Gy cesium-137 γ-rays. Data reported as mean ± SD; where error bars are not visible, they are smaller than the data point. Asterisks mark significant differences at *p*-values of ≤0.05 (*).

Supplementary Figure 3 Clonogenic survival of G0/G1-phase normal NFF28 primary fibroblasts **(A)** and asynchronously growing A549 lung carcinoma **(B)**, U2OS osteosarcoma **(C),** and U87MG malignant glioma cells **(D)** pretreated for 18 h with 1–20 µM concentrations of PTACH and exposed to 0.5–6 Gy cesium-137 γ-rays. Data reported as mean ± SD; where error bars are not visible, they are smaller than the data point. Asterisks mark significant differences at *p*-values of ≤0.05 (*).

Supplementary Figure 4 HDACi sensitizer enhancement ratio (SER) values ± SEM for G0/G1-phase normal NFF28 primary fibroblasts **(A)** and asynchronously growing A549 lung carcinoma **(B)**, U2OS osteosarcoma **(C)**, and U87MG malignant glioma cells **(D)** pretreated for 18 h with 1–20 µM concentrations of SAHA, M344, and PTACH and irradiated with cesium-137 γ-rays [calculated using D_10_ survival values; asterisks mark significant differences at *p*-values of ≤0.05 (*) and ≤0.01 (**)].

## Abbreviations

BER, base excision repair; CIRT, carbon ion radiation therapy; DDR, DNA damage response; DSB, double-strand break; DMSO, dimethyl sulfoxide; HDACi, histone deacetylase inhibitor; HRR, homologous recombinational repair; HZE, high *Z* and *E*; IMRT, intensity-modulated radiation therapy; M344, 4-(dimethylamino)-N-[7-(hydroxyamino)-7-oxoheptyl]-benzamide; NHEJ, non-homologous end-joining; PBRT, proton beam radiation therapy; PTACH, S-[6-(4-phenyl-2-thiazolylcarbamoyl)hexyl] thioisobutyrate; RBE, relative biological effectiveness; SAHA, suberoylanilide hydroxamic acid; SER, sensitizer enhancement ratio.
