# Supplementary figures and images for: Evaluation of Histone Deacetylase Inhibitors as Radiosensitizers for Proton and Light Ion Radiotherapy
Source: Front Oncol. 2021 Aug 26;11:735940. doi: 10.3389/fonc.2021.735940 (PMC8426582; doi:10.3389/fonc.2021.735940)

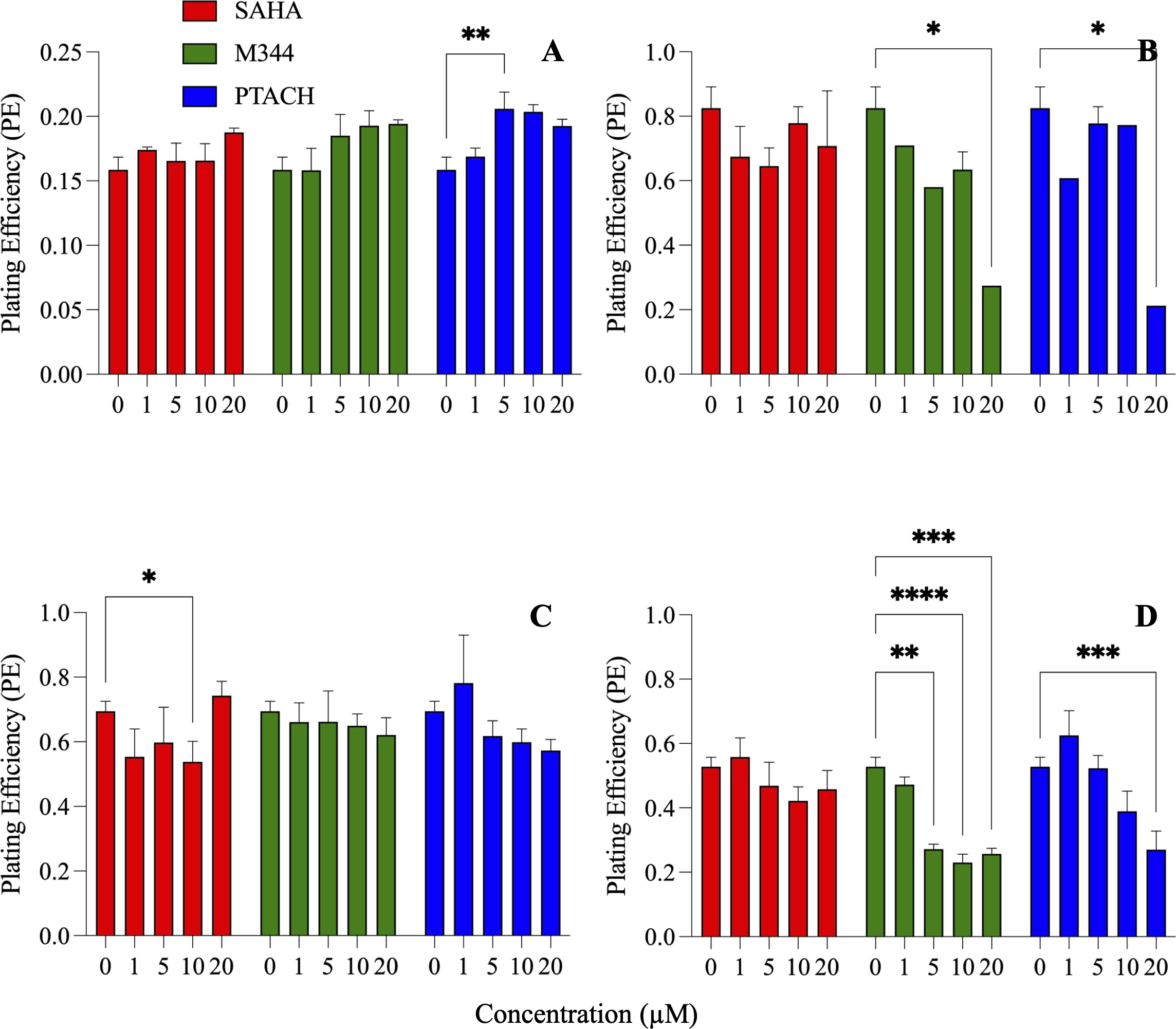

Supplement: Supplementary Figure 1 — Sham-irradiated plating efficiencies (PE) ± SEM for G0/G1-phase normal NFF28 primary fibroblasts (A) and asynchronously growing A549 lung carcinoma (B), U2OS osteosarcoma (C), and U87MG malignant glioma cells (D) pretreated for 18 h with 0.1% DMSO (vehicle control) or 1–20 µM concentrations of SAHA, M344, and PTACH. Asterisks mark significant differences at p-values of ≤0.05 (*), ≤0.01 (**), ≤0.001 (***), and ≤10−4 (****). [file Image_1.tiff]

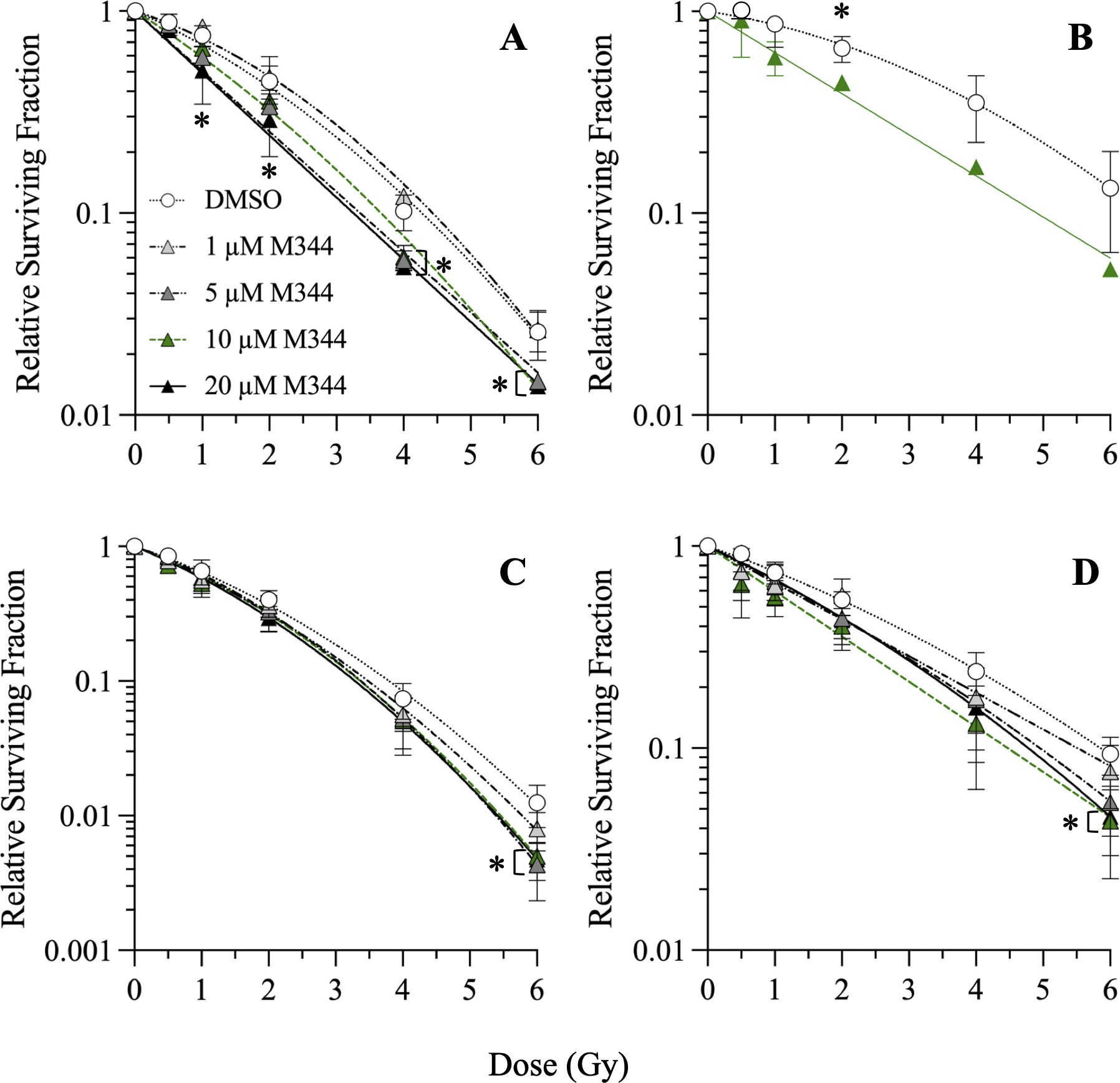

Supplement: Supplementary Figure 2 — Clonogenic survival of G0/G1-phase normal NFF28 primary fibroblasts (A) and asynchronously growing A549 lung carcinoma (B), U2OS osteosarcoma (C), and U87MG malignant glioma cells (D) pretreated for 18 h with 1–20 µM concentrations of M344 and exposed to 0.5–6 Gy cesium-137 γ-rays. Data reported as mean ± SD; where error bars are not visible, they are smaller than the data point. Asterisks mark significant differences at p-values of ≤0.05 (*). [file Image_2.tiff]

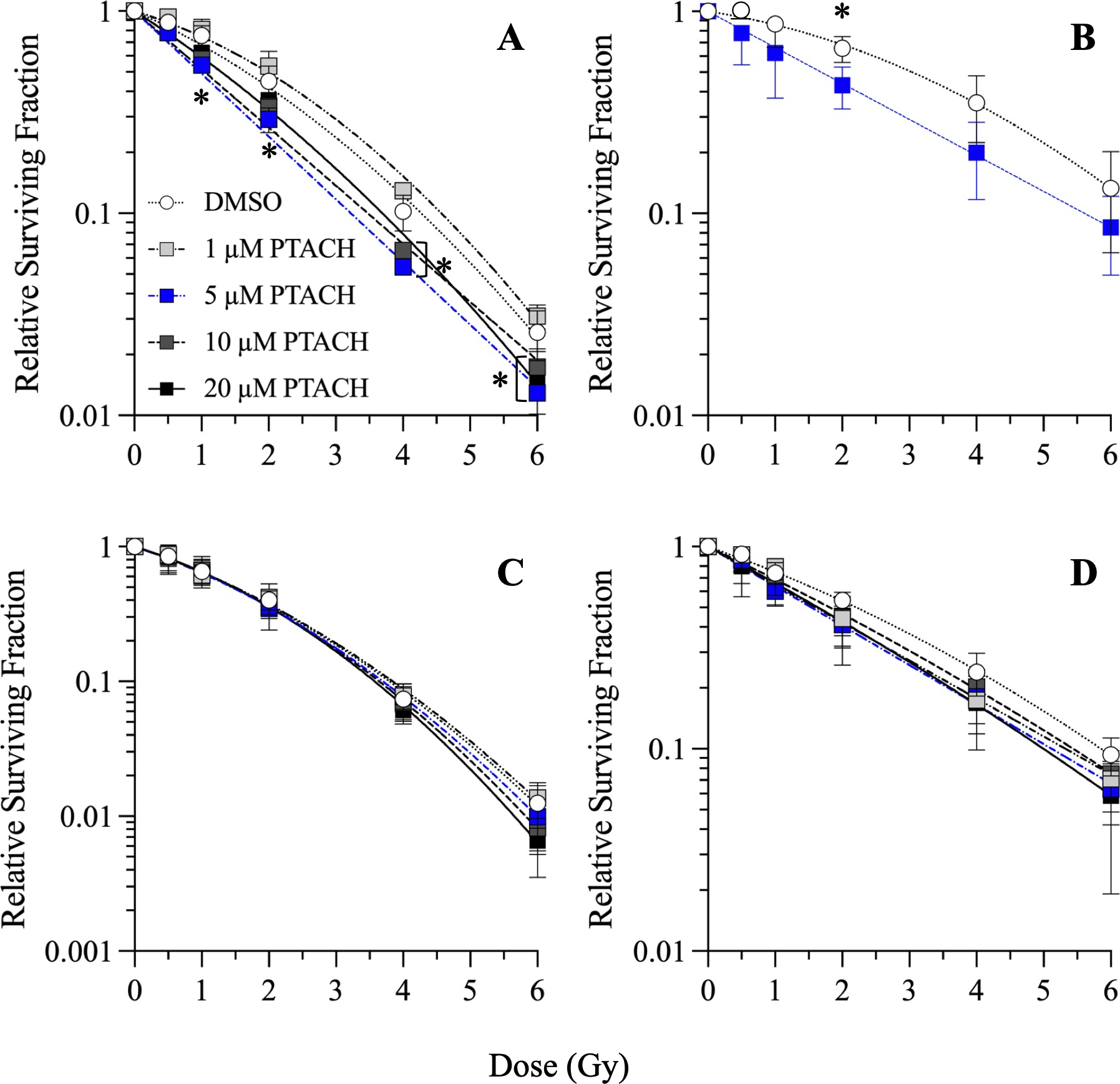

Supplement: Supplementary Figure 3 — Clonogenic survival of G0/G1-phase normal NFF28 primary fibroblasts (A) and asynchronously growing A549 lung carcinoma (B), U2OS osteosarcoma (C), and U87MG malignant glioma cells (D) pretreated for 18 h with 1–20 µM concentrations of PTACH and exposed to 0.5–6 Gy cesium-137 γ-rays. Data reported as mean ± SD; where error bars are not visible, they are smaller than the data point. Asterisks mark significant differences at p-values of ≤0.05 (*). [file Image_3.tiff]

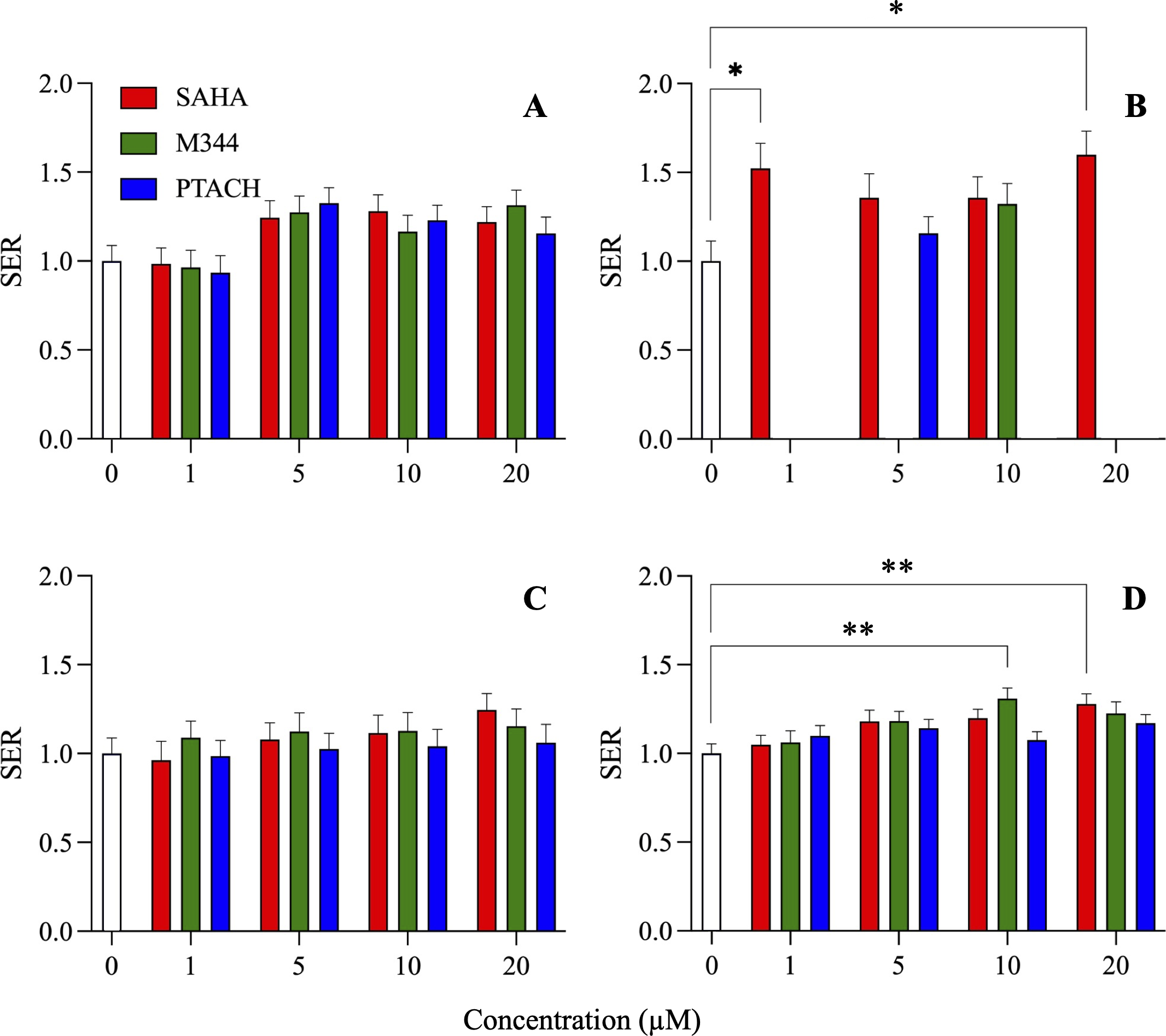

Supplement: Supplementary Figure 4 — HDACi sensitizer enhancement ratio (SER) values ± SEM for G0/G1-phase normal NFF28 primary fibroblasts (A) and asynchronously growing A549 lung carcinoma (B), U2OS osteosarcoma (C), and U87MG malignant glioma cells (D) pretreated for 18 h with 1–20 µM concentrations of SAHA, M344, and PTACH and irradiated with cesium-137 γ-rays [calculated using D10 survival values; asterisks mark significant differences at p-values of ≤0.05 (*) and ≤0.01 (**)]. [file Image_4.tiff]
