# Supplementary material for: Evaluation of Histone Deacetylase Inhibitors as Radiosensitizers for Proton and Light Ion Radiotherapy
Source: Front Oncol. 2021 Aug 26;11:735940. doi: 10.3389/fonc.2021.735940 (PMC8426582; doi:10.3389/fonc.2021.735940)
Supplement: Supplementary file 5 [file Table_1.docx]

**SI Table 1**. D_10_ (dose required for 10% relative survival) and HDACi sensitization enhancement ratio (SER) values for cesium-137 γ-ray irradiations of G0/G1-phase NFF28 apparently normal fibroblasts and asynchronously-growing A549 lung carcinoma, U2OS osteosarcoma, and U87MG malignant glioma cells (mean ± SEM).

| **Cell Line** | **HDACi** | **Concentration** | **D_10_ (Gy)** | **SER** |
| --- | --- | --- | --- | --- |
| NFF28 | Vehicle Control | 0.1% DMSO | 4.27 ± 0.26 | –– |
|  | SAHA | 1 µM | 4.34 ± 0.30 | 0.98 ± 0.09 |
|  |  | 5 µM | 3.43 ± 0.15 | 1.24 ± 0.09 |
|  |  | 10 µM | 3.34 ± 0.13 | 1.28 ± 0.09 |
|  |  | 20 µM | 3.51 ± 0.13 | 1.22 ± 0.09 |
|  | M344 | 1 µM | 4.43 ± 0.36 | 0.96 ± 0.10 |
|  |  | 5 µM | 3.35 ± 0.13 | 1.27 ± 0.09 |
|  |  | 10 µM | 3.66 ± 0.18 | 1.17 ± 0.09 |
|  |  | 20 µM | 3.25 ± 0.07 | 1.31 ± 0.09 |
|  | PTACH | 1 µM | 4.57 ± 0.38 | 0.93 ± 0.10 |
|  |  | 5 µM | 3.22 ± 0.07 | 1.33 ± 0.09 |
|  |  | 10 µM | 3.48 ± 0.11 | 1.23 ± 0.09 |
|  |  | 20 µM | 3.69 ± 0.18 | 1.16 ± 0.09 |
| A549 | Vehicle Control | 0.1% DMSO | 6.50 ± 0.52 | –– |
|  | SAHA | 1 µM | 4.27 ± 0.20 | 1.52 ± 0.14 |
|  |  | 5 µM | 4.78 ± 0.28 | 1.36 ± 0.13 |
|  |  | 10 µM | 4.78 ± 0.17 | 1.36 ± 0.12 |
|  |  | 20 µM | 4.06 ± 0.10 | 1.60 ± 0.13 |
|  | M344 | 10 µM | 4.91 ± 0.16 | 1.32 ± 0.11 |
|  | PTACH | 5 µM | 5.61 ± 0.07 | 1.16 ± 0.09 |
| U2OS | Vehicle Control | 0.1% DMSO | 3.80 ± 0.24 | –– |
|  | SAHA | 1 µM | 3.94 ± 0.35 | 0.96 ± 0.11 |
|  |  | 5 µM | 3.52 ± 0.22 | 1.08 ± 0.09 |
|  |  | 10 µM | 3.40 ± 0.22 | 1.12 ± 0.10 |
|  |  | 20 µM | 3.05 ± 0.12 | 1.25 ± 0.09 |
|  | M344 | 1 µM | 3.48 ± 0.20 | 1.09 ± 0.09 |
|  |  | 5 µM | 3.38 ± 0.23 | 1.12 ± 0.10 |
|  |  | 10 µM | 3.37 ± 0.23 | 1.13 ± 0.10 |
|  |  | 20 µM | 3.29 ± 0.18 | 1.15 ± 0.10 |
|  | PTACH | 1 µM | 3.85 ± 0.24 | 0.99 ± 0.09 |
|  |  | 5 µM | 3.70 ± 0.22 | 1.03 ± 0.09 |
|  |  | 10 µM | 3.64 ± 0.24 | 1.04 ± 0.09 |
|  |  | 20 µM | 3.58 ± 0.26 | 1.06 ± 0.10 |
| U87MG | Vehicle Control | 0.1% DMSO | 5.86 ± 0.22 | –– |
|  | SAHA | 1 µM | 5.58 ± 0.19 | 1.05 ± 0.05 |
|  |  | 5 µM | 4.96 ± 0.19 | 1.18 ± 0.06 |
|  |  | 10 µM | 4.88 ± 0.09 | 1.20 ± 0.05 |
|  |  | 20 µM | 4.58 ± 0.11 | 1.28 ± 0.06 |
|  | M344 | 1 µM | 5.51 ± 0.26 | 1.06 ± 0.06 |
|  |  | 5 µM | 4.95 ± 0.14 | 1.18 ± 0.06 |
|  |  | 10 µM | 4.47 ± 0.11 | 1.31 ± 0.06 |
|  |  | 20 µM | 4.78 ± 0.18 | 1.23 ± 0.07 |
|  | PTACH | 1 µM | 5.33 ± 0.20 | 1.10 ± 0.06 |
|  |  | 5 µM | 5.12 ± 0.10 | 1.14 ± 0.05 |
|  |  | 10 µM | 5.44 ± 0.10 | 1.08 ± 0.05 |
|  |  | 20 µM | 5.00 ± 0.08 | 1.17 ± 0.05 |
