# Supplementary material for: Evaluation of Histone Deacetylase Inhibitors as Radiosensitizers for Proton and Light Ion Radiotherapy
Source: Front Oncol. 2021 Aug 26;11:735940. doi: 10.3389/fonc.2021.735940 (PMC8426582; doi:10.3389/fonc.2021.735940)
Supplement: Supplementary file 6 [file Table_2.docx]

**SI Table 2**. D_10_ (dose required for 10% relative survival), relative biological effectiveness (RBE) and HDACi sensitization enhancement ratio (SER) values for Bragg plateau 250 MeV proton, 290 MeV/n C-12 ion and 350 MeV/n O-16 ion irradiations (relative to cesium-137 γ-ray irradiations) of G0/G1-phase NFF28 apparently normal fibroblasts and asynchronously-growing A549 lung carcinoma, U2OS osteosarcoma, and U87MG malignant glioma cells (mean ± SEM).

| **Cell Line** | **IR** | **[HDACi]** | **D_10_ (Gy)** | **RBE** | **SER** |
| --- | --- | --- | --- | --- | --- |
| NFF28 | 250 MeV  Protons | 0.1% DMSO | 4.15 ± 0.24 | 1.03 ± 0.09 | –– |
|  |  | 10 µM SAHA | 3.61 ± 0.18 | 0.93 ± 0.06 | 1.15 ± 0.09 |
|  |  | 10 µM M344 | 3.52 ± 0.18 | 1.04 ± 0.07 | 1.18 ± 0.09 |
|  |  | 5 µM PTACH | 3.58 ± 0.21 | 0.90 ± 0.06 | 1.16 ± 0.10 |
|  | 290 MeV/n  C-12 ions | 0.1% DMSO | 3.31 ± 0.10 | 1.29 ± 0.09 | –– |
|  |  | 10 µM SAHA | 4.08 ± 0.07 | 0.82 ± 0.03 | 0.81 ± 0.03 |
|  |  | 10 µM M344 | 4.01 ± 0.08 | 0.91 ± 0.05 | 0.83 ± 0.03 |
|  |  | 5 µM PTACH | 3.76 ± 0.09 | 0.86 ± 0.03 | 0.88 ± 0.03 |
|  | 350 MeV/n  O-16 ions | 0.1% DMSO | 2.72 ± 0.05 | 1.57 ± 0.10 | –– |
|  |  | 10 µM SAHA | 4.10 ± 0.09 | 0.81 ± 0.04 | 0.66 ± 0.02 |
|  |  | 10 µM M344 | 4.22 ± 0.08 | 0.87 ± 0.04 | 0.64 ± 0.04 |
|  |  | 5 µM PTACH | 4.57 ± 0.12 | 0.71 ± 0.02 | 0.60 ± 0.04 |
| A549 | 250 MeV  Protons | 0.1% DMSO | 7.00 ± 0.40 | 0.93 ± 0.09 | –– |
|  |  | 10 µM SAHA | 5.64 ± 0.45 | 0.85 ± 0.07 | 1.24 ± 0.12 |
|  |  | 10 µM M344 | 7.85 ± 0.53 | 0.63 ± 0.05 | 0.89 ± 0.08 |
|  |  | 5 µM PTACH | 11.23 ± 1.69 | 0.50 ± 0.08 | 0.62 ± 0.10 |
|  | 290 MeV/n  C-12 ions | 0.1% DMSO | 2.96 ± 0.07 | 2.19 ± 0.18 | –– |
|  |  | 10 µM SAHA | 2.76 ± 0.05 | 1.73 ± 0.07 | 1.07 ± 0.03 |
|  |  | 10 µM M344 | 2.62 ± 0.07 | 1.87 ± 0.08 | 1.13 ± 0.04 |
|  |  | 5 µM PTACH | 3.02 ± 0.12 | 1.86 ± 0.07 | 0.98 ± 0.04 |
|  | 350 MeV/n  O-16 ions | 0.1% DMSO | 4.61 ± 0.15 | 1.41 ± 0.12 | –– |
|  |  | 10 µM SAHA | 5.15 ± 0.36 | 0.93 ± 0.07 | 0.89 ± 0.07 |
|  |  | 10 µM M344 | 5.39 ± 0.35 | 0.91 ± 0.07 | 0.86 ± 0.09 |
|  |  | 5 µM PTACH | 4.83 ± 0.19 | 1.16 ± 0.05 | 0.95 ± 0.09 |
| U2OS | 250 MeV  Protons | 0.1% DMSO | 3.66 ± 0.34 | 1.04 ± 0.12 | –– |
|  |  | 10 µM SAHA | 3.59 ± 0.11 | 0.95 ± 0.07 | 1.02 ± 0.10 |
|  |  | 10 µM M344 | 3.81 ± 0.15 | 0.88 ± 0.07 | 0.96 ± 0.10 |
|  |  | 5 µM PTACH | 3.94 ± 0.24 | 0.94 ± 0.08 | 0.93 ± 0.10 |
|  | 290 MeV/n  C-12 ions | 0.1% DMSO | 2.17 ± 0.06 | 1.75 ± 0.12 | –– |
|  |  | 10 µM SAHA | 2.39 ± 0.14 | 1.42 ± 0.12 | 0.91 ± 0.06 |
|  |  | 10 µM M344 | 2.27 ± 0.12 | 1.48 ± 0.13 | 0.95 ± 0.06 |
|  |  | 5 µM PTACH | 2.43 ± 0.18 | 1.52 ± 0.14 | 0.89 ± 0.07 |
|  | 350 MeV/n  O-16 ions | 0.1% DMSO | 2.54 ± 0.12 | 1.50 ± 0.12 | –– |
|  |  | 10 µM SAHA | 2.20 ± 0.06 | 1.55 ± 0.11 | 1.15 ± 0.06 |
|  |  | 10 µM M344 | 2.20 ± 0.08 | 1.53 ± 0.12 | 1.15 ± 0.08 |
|  |  | 5 µM PTACH | 2.34 ± 0.07 | 1.58 ± 0.10 | 1.08 ± 0.07 |
| U87G | 250 MeV  Protons | 0.1% DMSO | 7.77 ± 0.54 | 0.75 ± 0.06 | –– |
|  |  | 10 µM SAHA | 6.82 ± 0.56 | 0.72 ± 0.06 | 1.14 ± 0.12 |
|  |  | 10 µM M344 | 6.75 ± 0.43 | 0.66 ± 0.05 | 1.15 ± 0.11 |
|  |  | 5 µM PTACH | 5.37 ± 0.09 | 0.95 ± 0.02 | 1.45 ± 0.10 |
|  | 290 MeV/n  C-12 ions | 0.1% DMSO | 3.47 ± 0.28 | 1.69 ± 0.15 | –– |
|  |  | 10 µM SAHA | 3.96 ± 0.35 | 1.23 ± 0.11 | 0.88 ± 0.10 |
|  |  | 10 µM M344 | 2.91 ± 0.10 | 1.54 ± 0.06 | 1.19 ± 0.10 |
|  |  | 5 µM PTACH | 2.93 ± 0.14 | 1.75 ± 0.09 | 1.18 ± 0.11 |
|  | 350 MeV/n  O-16 ions | 0.1% DMSO | 3.70 ± 0.09 | 1.58 ± 0.07 | –– |
|  |  | 10 µM SAHA | 3.15 ± 0.11 | 1.55 ± 0.06 | 1.18 ± 0.05 |
|  |  | 10 µM M344 | 3.29 ± 0.09 | 1.36 ± 0.05 | 1.13 ± 0.05 |
|  |  | 5 µM PTACH | 3.10 ± 0.09 | 1.65 ±0.06 | 1.19 ± 0.06 |
