# Supplementary material for: Evaluation of Histone Deacetylase Inhibitors as Radiosensitizers for Proton and Light Ion Radiotherapy
Source: Front Oncol. 2021 Aug 26;11:735940. doi: 10.3389/fonc.2021.735940 (PMC8426582; doi:10.3389/fonc.2021.735940)
Supplement: Supplementary file 7 [file Table_3.docx]

**SI Table 3**. D_10_ (dose required for 10% relative survival), relative biological effectiveness (RBE) and HDACi sensitization enhancement ratio (SER) values for cesium-137 γ-ray and Bragg plateau 250 MeV proton, 290 MeV/n C-12 ion and 350 MeV/n O-16 ion irradiations of asynchronously-growing (log-phase) NFF28 apparently normal fibroblasts (mean ± SEM).

| **IR** | **[HDACi]** | **D_10_ (Gy)** | **RBE** | **SER** |
| --- | --- | --- | --- | --- |
| Cesium-137  γ-rays | 0.1% DMSO | 6.78 ± 1.10 | N/A | –– |
|  | 10 µM SAHA | 6.00 ± 0.48 |  | 1.13 ± 0.18 |
|  | 10 µM M344 | 5.96 ± 0.74 |  | 1.14 ± 0.20 |
|  | 5 µM PTACH | 5.52 ± 0.74 |  | 1.23 ± 0.21 |
| 250 MeV  Protons | 0.1% DMSO | 5.72 ± 0.16 | 1.19 ± 0.16 | –– |
|  | 10 µM SAHA | 4.39 ± 0.23 | 1.37 ± 0.10 | 1.30 ± 0.06 |
|  | 10 µM M344 | 4.73 ± 0.36 | 1.26 ± 0.15 | 1.21 ± 0.08 |
|  | 5 µM PTACH | 4.56 ± 0.13 | 1.21 ± 0.14 | 1.25 ± 0.04 |
| 290 MeV/n  C-12 ions | 0.1% DMSO | 3.82 ± 0.31 | 1.77 ± 0.18 | –– |
|  | 10 µM SAHA | 3.23 ± 0.06 | 1.86 ± 0.08 | 1.18 ± 0.08 |
|  | 10 µM M344 | 3.28 ± 0.12 | 1.82 ± 0.13 | 1.17 ± 0.09 |
|  | 5 µM PTACH | 3.64 ± 0.15 | 1.52 ± 0.14 | 1.05 ± 0.09 |
| 350 MeV/n  O-16 ions | 0.1% DMSO | 4.39 ± 0.27 | 1.55 ± 0.17 | –– |
|  | 10 µM SAHA | 2.90 ± 0.24 | 2.07 ± 0.12 | 1.51 ± 0.10 |
|  | 10 µM M344 | 3.81 ± 0.13 | 1.56 ± 0.13 | 1.15 ± 0.07 |
|  | 5 µM PTACH | 3.56 ± 0.24 | 1.55 ± 0.15 | 1.23 ± 0.09 |
