# Supplementary material for: Evaluation of Histone Deacetylase Inhibitors as Radiosensitizers for Proton and Light Ion Radiotherapy
Source: Front Oncol. 2021 Aug 26;11:735940. doi: 10.3389/fonc.2021.735940 (PMC8426582; doi:10.3389/fonc.2021.735940)
Supplement: Supplementary file 8 [file Table_4.docx]

**SI Table 4**. Yields of IR-induced anchorage-independent NFF28 fibroblast transformants per 10^5^ surviving cells•Gy^–1^, relative biological effectiveness (RBE) and HDACi sensitization enhancement ratio (SER) values for cesium-137 γ-ray and Bragg plateau 250 MeV proton, 290 MeV/n C-12 ion and 350 MeV/n O-16 ion irradiations of asynchronously-growing (log-phase) NFF28 apparently normal fibroblasts (mean ± SEM).

| **Dose Range** | **IR** | **[HDACi]** | **Transformants/**  **10^5^ surviving cells•Gy^–1^** | **RBE** | **SER** |
| --- | --- | --- | --- | --- | --- |
|  | Cesium-137  γ-rays | 0.1% DMSO | 247.2 ± 47.7 | N/A | –– |
|  |  | 10 µM SAHA | 210.4 ± 21.5 |  | 0.85 ± 0.22 |
| Low Dose  (≤25 cGy) |  | 10 µM M344 | 215.8 ± 51.6 |  | 0.87 ± 0.31 |
|  |  | 5 µM PTACH | 148.3 ± 1.9 |  | 0.60 ± 0.19 |
|  | 250 MeV  Protons | 0.1% DMSO | 319.4 ± 26 | 1.29 ± 0.21 | –– |
|  |  | 10 µM SAHA | 263.2 ± 31.5 | 1.25 ± 0.16 | 0.82 ± 0.14 |
|  |  | 10 µM M344 | 192.7 ± 9.9 | 0.89 ± 0.24 | 0.60 ± 0.10 |
|  |  | 5 µM PTACH | 286.8 ± 5.4 | 1.93 ± 0.02 | 0.90 ± 0.08 |
|  | 290 MeV/n  C-12 ions | 0.1% DMSO | 274.3 ± 63.9 | 1.11 ± 0.30 | –– |
|  |  | 10 µM SAHA | 210.5 ± 22.1 | 1.00 ± 0.15 | 0.77 ± 0.26 |
|  |  | 10 µM M344 | 195.9 ± 19.6 | 0.91 ± 0.26 | 0.71 ± 0.25 |
|  |  | 5 µM PTACH | 325.7 ± 75.3 | 2.20 ± 0.23 | 1.19 ± 0.33 |
|  | 350 MeV/n  O-16 ions | 0.1% DMSO | 338.5 ± 56.1 | 1.37 ± 0.25 | –– |
|  |  | 10 µM SAHA | 361.3 ± 86.9 | 1.72 ± 0.26 | 1.07 ± 0.29 |
|  |  | 10 µM M344 | 270.1 ± 61.3 | 1.25 ± 0.33 | 0.80 ± 0.28 |
|  |  | 5 µM PTACH | 340.5 ± 124.3 | 2.30 ± 0.37 | 1.01 ± 0.40 |
| High Dose  (100 cGy) | Cesium-137  γ-rays | 0.1% DMSO | 97.1 ± 23.8 | N/A | –– |
|  |  | 10 µM SAHA | 114.9 ± 27.1 |  | 1.18 ± 0.34 |
|  |  | 10 µM M344 | 131.2 ± 30.9 |  | 1.35 ± 0.34 |
|  |  | 5 µM PTACH | 112.3 ± 31.8 |  | 1.16 ± 0.37 |
|  | 250 MeV  Protons | 0.1% DMSO | 71.4 ± 18.8 | 0.74 ± 0.36 | –– |
|  |  | 10 µM SAHA | 80.9 ± 17 | 0.70 ± 0.32 | 1.13 ± 0.34 |
|  |  | 10 µM M344 | 46.6 ± 14.1 | 0.36 ± 0.38 | 0.65 ± 0.40 |
|  |  | 5 µM PTACH | 79.8 ± 20.4 | 0.71 ± 0.38 | 1.12 ± 0.37 |
|  | 290 MeV/n  C-12 ions | 0.1% DMSO | 163.4 ± 29.6 | 1.68 ± 0.30 | –– |
|  |  | 10 µM SAHA | 206.3 ± 31.6 | 1.80 ± 0.28 | 1.26 ± 0.24 |
|  |  | 10 µM M344 | 179.8 ± 24.5 | 1.37 ± 0.27 | 1.10 ± 0.23 |
|  |  | 5 µM PTACH | 267.7 ± 46.3 | 2.38 ± 0.33 | 1.64 ± 0.25 |
|  | 350 MeV/n  O-16 ions | 0.1% DMSO | 314 ± 80 | 3.23 ± 0.35 | –– |
|  |  | 10 µM SAHA | 274.2 ± 74.8 | 2.39 ± 0.36 | 0.87 ± 0.37 |
|  |  | 10 µM M344 | 454.4 ± 99.9 | 3.46 ± 0.32 | 1.45 ± 0.34 |
|  |  | 5 µM PTACH | 417.3 ± 112.8 | 3.72 ± 0.39 | 1.33 ± 0.37 |
